# Hidden β-γ Dehydrogenation Products in Long-Chain Fatty Acid Oxidation Unveiled by NMR: Implications on Lipid Metabolism

**DOI:** 10.1021/acsbiomedchemau.4c00140

**Published:** 2025-03-15

**Authors:** Simone Fabbian, Beatrice Masciovecchio, Elisabetta Schievano, Gabriele Giachin

**Affiliations:** # Department of Chemical Sciences, University of Padua, via F. Marzolo 1, 35131 Padova, Italy; § Department of Pharmaceutical and Pharmacological Sciences, University of Padua, via F. Marzolo 5, 35131 Padova, Italy

**Keywords:** palmitoyl-CoA, fatty acid β-oxidation, NMR, ACAD9, VLCAD

## Abstract

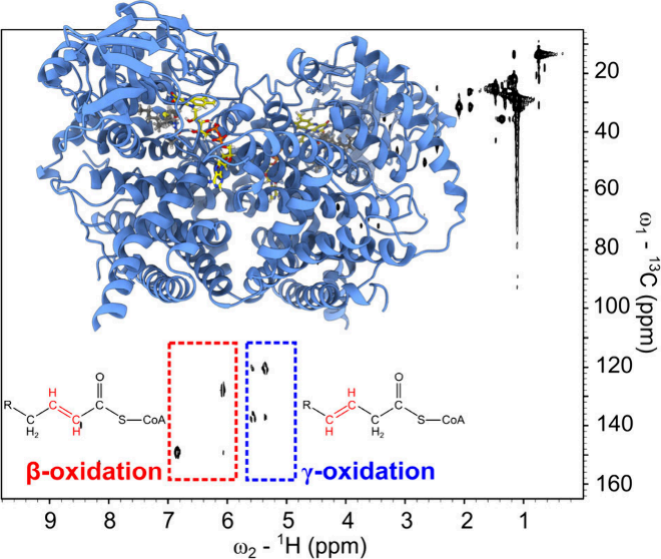

We present a comprehensive analysis of the initial α,β-dehydrogenation step in long-chain fatty acid β-oxidation (FAO). We focused on palmitoyl-CoA oxidized by two mitochondrial acyl-CoA dehydrogenases, very-long-chain acyl-CoA dehydrogenase (VLCAD) and acyl-CoA dehydrogenase family member 9 (ACAD9), both implicated in mitochondrial diseases. By combining MS and NMR, we identified the (2*E*)-hexadecenoyl-CoA as the expected α-β-dehydrogenation product and also the *E* and *Z* stereoisomers of 3-hexadecenoyl-CoA: a “γ-oxidation” product. This finding reveals an alternative catalytic pathway in mitochondrial FAO, suggesting a potential regulatory role for ACAD9 and VLCAD during fatty acid metabolism.

Mitochondrial fatty acid β-oxidation (FAO) plays a critical role in cellular energy metabolism.^1,2^ Disruptions in this process lead to serious metabolic disorders, most notably VLCAD (Very Long-Chain Acyl-CoA Dehydrogenase) and ACAD9 (Acyl-CoA Dehydrogenase 9) deficiencies.^3^ VLCAD deficiency manifests as a multiorgan disease; ACAD9 plays a dual role in both FAO and mitochondrial Complex I assembly,^4^ and ACAD9 deficiency mainly results in neurological symptoms (e.g., Leigh’s disease).^5^ FAO defects are also linked to dysfunctions in oxidative phosphorylation system, as both pathways are connected in mitochondrial energy metabolism.^6^ Treatment for VLCAD and ACAD9 (hereafter ACAD) deficiencies are limited to supportive care with no available cure.^7^

In FAO, the reaction begins with the dehydrogenation of an acyl-CoA substrate at its α- and β-carbons. Within the substrate binding site, a conserved glutamate at position 426 (hereafter in ACAD9 numbering) acts as catalytic base and it abstracts a proton from the α-carbon, while nitrogen at position 5 of the isoalloxazine ring within the FAD cofactor facilitates the removal of a hydride from the β-carbon (Scheme 1). This coordinated action results in the formation of a *trans* double bond between α and β-carbons.^8^ The studies on FAO have relied mainly on florescence-based assays, focusing on the canonical α,β-dehydrogenation catalyzed by MCAD (Medium-Chain Acyl-CoA Dehydrogenase).^9,10^ These techniques have limitations, particularly in differentiating noncanonical isomeric intermediates. Advances in NMR spectroscopy hold promise for unraveling these more complex enzymatic activities. Despite the progress in understanding the FAO of short-chain fatty acid oxidation, our knowledge of long-chain (LC) β-oxidation remains limited. This gap is significant, as it prevents us from comprehending the molecular determinants underlying ACAD deficiencies.^11^

**Scheme 1:**
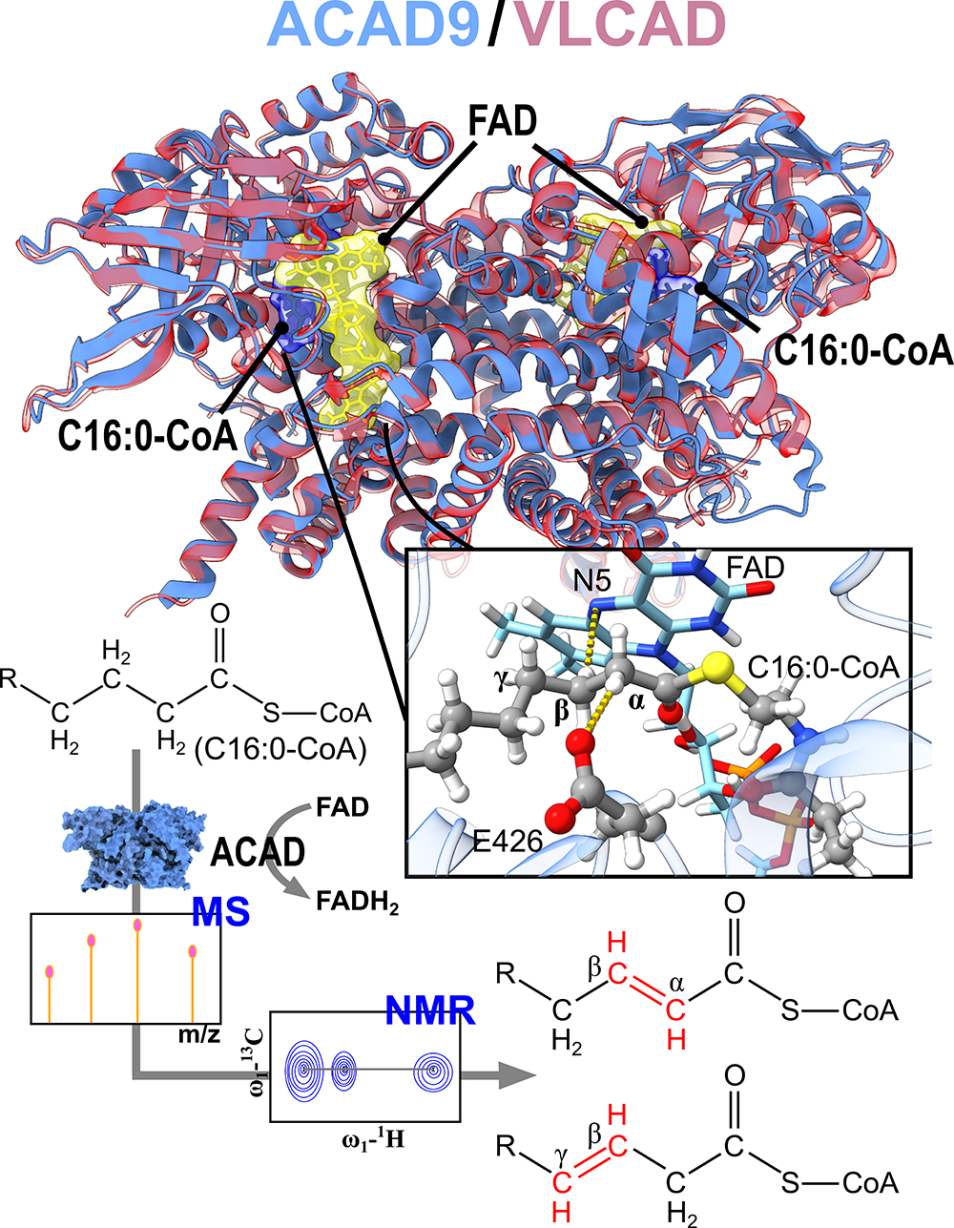
Experimental Design and Mechanistic overview of ACAD-Mediated FAO Superposition of VLCAD (in red) and ACAD9 (in blue) homodimers. The positions of FAD cofactor and palmitoyl-CoA (C16:0-CoA) substrate are highlighted. Inset shows key interactions between substrate and the active site. MS and NMR approaches were employed to analyze α-β and noncanonical β-γ dehydrogenation products.

Our research addresses the gap in understanding LC FAO, specifically focusing on VLCAD and ACAD9: two enzymes sharing conserved folding and overlapping functions.^12^ We explored the dehydrogenation of LC fatty acids revealing unexplored dehydrogenation products besides the canonical β-oxidation reaction. Our previous studies on these enzymes shed light into their structural features.^12^ Here, we first provide evidence that the recombinant ACAD enzymes are pure, contain FAD (Figure 1A, Figure S1), and are enzymatically active, as demonstrated by their ability to transfer electrons to electron-transfer flavoprotein (ETF). This activity was confirmed through ETF fluorescence reduction experiments (Figure 1B,C and Figure S2) using palmitoyl-CoA (C16:0-CoA, Figure S3A) as the canonical substrate.^13,14^ This assay monitors the fluorescence quenching of ETF upon electron transfer from ACAD.^15^ The mean activities for ACAD9 and VLCAD are 131 ± 34 and 163 ± 30 mU/mg, respectively.

**Figure 1:**
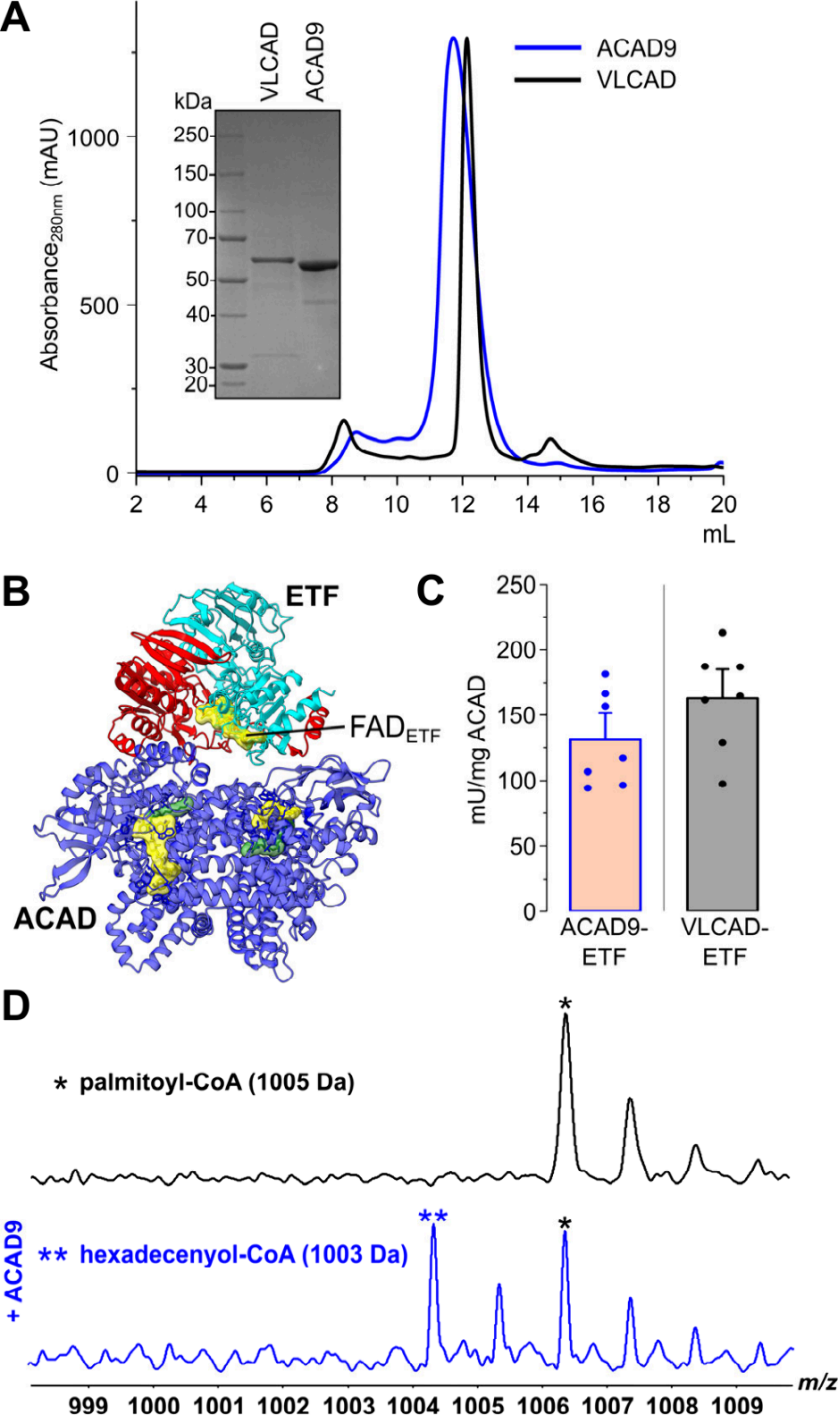
C16:0-CoA β-oxidation was monitored by fluorescence and MS. (**A**) SEC traces of VLCAD and ACAD9 (black and blue lines, respectively) and SDS-PAGE showing protein monodispersity and purity. (**B**) AlphaFold model of the ACAD9 homodimer (in blue) in complex with ETF. (**C**) Mean enzyme activities for ACAD9 and VLCAD obtained from replicate assays. (**D**) The upper MS spectrum identifies palmitoyl-CoA in the absence of ACAD9. The lower spectrum in the presence of ACAD9 (blue) shows an additional product at 1003 Da identified as hexadecenoyl-CoA.

To obtain a more detailed understanding of the dehydrogenation mechanism and to identify the exact products formed, we turned to MS and NMR spectroscopy approaches to analyze the β-oxidation of C16:0-CoA. The structural integrity of C16:0-CoA was confirmed through a combination of MS and NMR spectroscopy experiments (Figures S3, S4 and S14, Table S1 and S6).

To ensure the optimal detection of dehydrogenation products in the MS analysis, we employed an enzyme-to-substrate molar ratio of ∼ 1:30. The substrate concentration was kept below the critical micelle concentration.^16^ Interrogation of β-oxidation on C16:0-CoA in the presence of ACAD9 enables the identification of a single desaturation product of 1003 Da, canonically attributed to (2*E*)-hexadecenoyl-CoA (Figure 1D and Figure S5, Table S2). This result is also consistent in the presence of VLCAD (Figure S6 and Table S3). Notably, both ACAD9 and VLCAD are shown to continue processing C16:0-CoA in the absence of ETF, which functions as physiological electron acceptor. Previous studies proposed that residual O_2_ present in the experimental setup can act as an alternative electron acceptor^17,18^ facilitating the reoxidation of FADH_2_ to FAD. This O_2_-mediated reoxidation process explains why incomplete β-oxidation is observed, as demonstrated by MS data showing both the product and unreacted substrate (Figure 1D).

To gain deeper structural insights, we turned to NMR spectroscopy. First, the resonances correlating ^1^H–^13^C (Figure 2A) were assigned to distinct the four molecular regions of the palmitoyl-CoA, **(1)**.^19,20^ The HSQC spectra obtained in the presence of both enzymes were nearly identical; for this reason, the NMR spectra obtained from the substrate incubated with ACAD9 are shown in the main text, while those with VLCAD are shown in the supporting materials (Figure S7). By comparing the HSQC spectra of ^13^C_16_-labeled C16:0-CoA before and after incubation with ACAD9 or VLCAD, we were able to identify the formation of different desaturation products. The canonical α,β-dehydrogenation reaction can be observed by the chemical shifts in the ^1^H–^13^C spectra, corresponding to the conjugated double bond formed during the enzymatic reaction. These cross-peaks occur in the typical regions for such unsaturated systems, with ^1^H signals appearing between 6 and 7 ppm and ^13^C signals between 120 and 155 ppm (Table S8). This pattern is distinctly highlighted in the red box in Figure 2B. This was further confirmed in ^1^H–^1^H TOCSY spectrum showing correlations between the α and β-protons and their connectivity to the methylene groups at positions 4, 5, and 6 of the substrate (Figure 3A).

**Figure 2:**
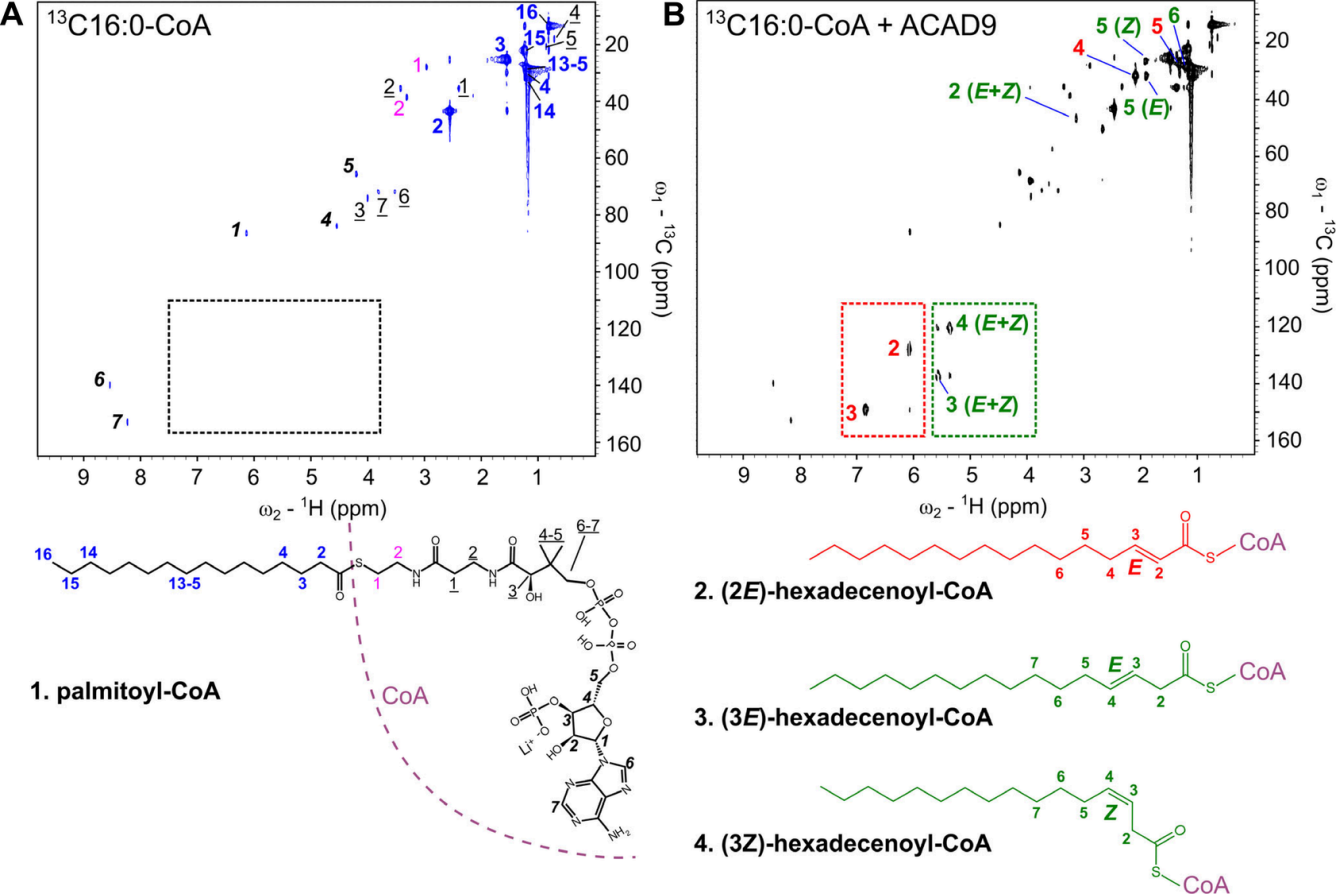
Identification of α,β and β,γ-desaturation products of C16:0-CoA by ACAD9. (**A**) HSQC spectrum of ^13^C_16_-labeled C16:0-CoA (**1**) before incubation with ACAD9, showing resonance assignments for distinct molecular regions. Key assignments: blue for palmitic acid, purple for cysteamine, underlined for pantothenic acid, and in bold for 3′-phosphoadenosine-5′-diphosphate. Black dotted box indicates the absence of ^1^H signals between 6 and 7 ppm and ^13^C signals between 120 and 155 ppm. (**B**) HSQC spectrum of the substrate after incubation with ACAD9 highlighting newly formed desaturation products. The red dotted box indicates the characteristic α,β-dehydrogenation product (2*E*)-hexadecenoyl-CoA (**2**, in red). In the green box: additional signals between 5.4 and 5.7 ppm in the ^1^H dimension and 120–140 ppm in the ^13^C dimension suggest an alternative double bond, leading to (3*E*)-hexadecenoyl-CoA (**3**) and (3*Z*)-hexadecenoyl-CoA (**4**).

**Figure 3:**
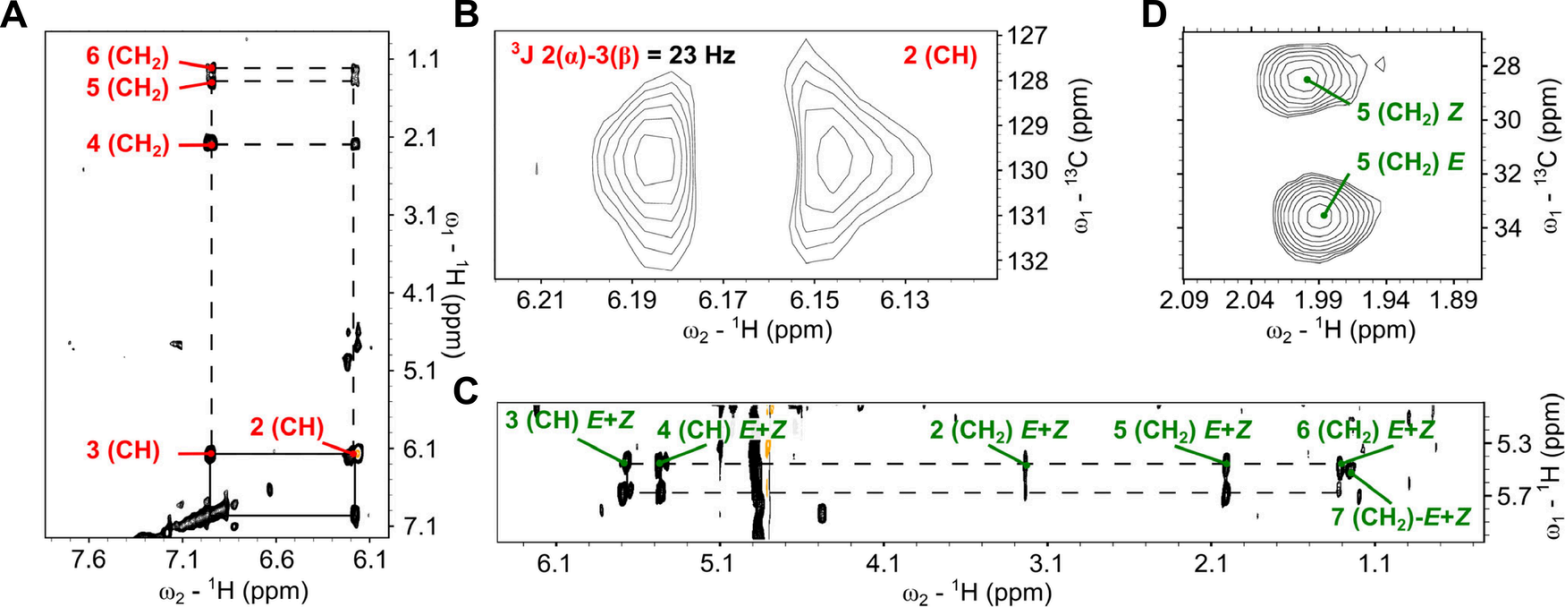
Structural characterization of α,β and β,γ dehydrogenation products by ACAD9. (**A**) Expanded region of the ^1^H–^1^H TOCSY spectrum, showing key correlations between protons along the acyl chain of product **(2)**; (**B**) Detailed HSQC spectrum section showing the splitting of the α-proton signal into a doublet confirming the *E*-configuration of product **(2)**; (**C**) TOCSY correlations between the protons of the new double bond in β-γ positions and the first four methylene groups of the C16:0-CoA chain after incubation with ACAD9, confirming the formation of products **(3)** and **(4)**; (**D**) HSQC spectrum displaying two distinct cross-peaks for the allylic methylene group of products **(3)** and **(4)** indicating the presence of both *E* and *Z* isomers.

We also observed structural details by analyzing the splitting of the α-proton signal in the HSQC spectrum (Figure 3B). The signal appears as a doublet (^3^J-coupling constant of ∼20 Hz) confirming the *E*-configuration of the double bond in the product (**2**). Most notably, additional signals were detected in the olefinic region of the HSQC spectrum between ^1^H 5.4 and 5.7 ppm and between ^13^C 120–140 ppm (Figure 2B, green box). Identical signals are visible in the HSQC spectrum of C16:0-CoA processed by VLCAD (Figure S7). This observation led us to hypothesize the formation of a second, alternative double bond along the acyl chain, indicative of previously uncharacterized enzymatic activity of these enzymes. Like the canonical α,β double bond, the cross-peaks associated with the C–H of this new double bond are distinctly separated in both the ^1^H and ^13^C dimensions, indicating a location close to the carbonyl group.

The TOCSY correlation of these novel olefin protons (Figure 3C) displays a pronounced deshielding signal at 3.22 ppm, which we assigned to methylene (Table S8) closest to the carbonyl. This implies the formation of a double bond in β-γ carbon positions. Interestingly, the allylic methylene group in position 5 associated with this β,γ double bond displayed two distinct cross-peaks in the HSQC spectrum, showing nearly identical ^1^H shifts (∼2 ppm) but differing ^13^C shifts (28.6 and 33.6 ppm).^21^ This pattern is consistent with the formation of both (3*E*)-hexadecenoic-CoA **(3)** and (3*Z*)-hexadecenoic-CoA stereoisomers **(4)** (Figure 3D).

The relative distribution of β- and γ-oxidized products is similar between ACAD9 and VLCAD, with both enzymes generating comparable proportions of the two oxidation products (Figure S8 A,B). Quantitative analysis of the HSQC spectra revealed a higher yield of the (3*E*) stereoisomer (∼66%) compared to that of the (3*Z*) stereoisomer (∼34%) when using ACAD9 or VLCAD (Figure S8 C,D). These findings suggest that both ACAD9 and VLCAD catalyze γ-oxidation with a similar overall efficiency while exhibiting a consistent stereochemical preference for the (3*E*) isomer.

To further validate the β,γ-dehydrogenation process, we conducted additional NMR experiments using (9*Z*)-C16:1-CoA (palmitoleoyl-CoA) a secondary substrate specific only for ACAD9^13^ (details on this substrate characterization in Figure S9, S10, and S15, Tables S4 and S7). Similarly to C16:0-CoA, following (9*Z*)-C16:1-CoA incubation with ACAD9, the MS spectrum revealed a single dehydrogenation event (Figure S11 and Table S5); the ^1^H–^1^H TOCSY spectrum enabled the characterization of the canonical α,β-dehydrogenated product (Figure S12A,B and Table S9).

Intriguingly, we detected also signals corresponding to the β,γ double bond formation in the 5.4–5.7 ppm region of the ^1^H spectrum (Figure S12C). All other ^1^H–^1^H correlations in the TOCSY spectrum matched those observed for C16:0-CoA after reaction with ACAD9, confirming the formation of both α,β and β,γ-dehydrogenated products (Figure S13). The unavailability of uniformly ^13^C-labeled C16:1-CoA precluded HSQC analysis, limiting the possibility of determining the stereochemistry of these products.

This is the first report of a β,γ-dehydrogenation, denoted by us γ-oxidation, occurring on LC fatty acids. We argue that this β,γ-dehydrogenation likely parallels the established α,β-dehydrogenation mechanism observed for MCAD. The α-carbon in acyl-CoA is activated via proton abstraction by an active site glutamate, which lowers the p*K*_a_ of the α-hydrogens and facilitates deprotonation.^9,10^ In VLCAD and ACAD9, which have larger binding pockets,^22,23^ a similar activation may involve also the β-position. Here, despite being inherently less acidic, the β-hydrogens may have a locally lowered p*K*_a_ due to interactions within the active site, rendering them available for deprotonation. Following this, the γ-hydrogen is transferred as a hydride to the isoalloxazine ring, leading to the formation of a double bond in position β,γ.

The discovery of γ-oxidation in LC fatty acids suggests an additional layer of complexity within FAO. The 2-enoyl-CoA hydratase (ECHS1), that selectively hydrates α,β-unsaturated enoyl-CoA produced by ACAD may not recognize noncanonical β,γ intermediates.^24^ These products require an isomerization step, typically catalyzed by enoyl-CoA isomerases (e.g., ECI1, ECI2 or ECH1) in order to proceed in the FAO cycle^25^ (Figure 4). Phylogenetic analysis confirms the evolutionary separation between ACADs and enoyl-CoA isomerases, reinforcing their distinct functional roles in fatty acid metabolism (Figure S16). The requirement for an additional isomerization step could influence the pathway rate by introducing a potential bottleneck. Such a mechanism suggests that β,γ-dehydrogenation activity could serve an adaptive regulatory role under specific metabolic conditions, adjusting FAO flux in response to changes in cellular demand or redox state.

**Figure 4:**
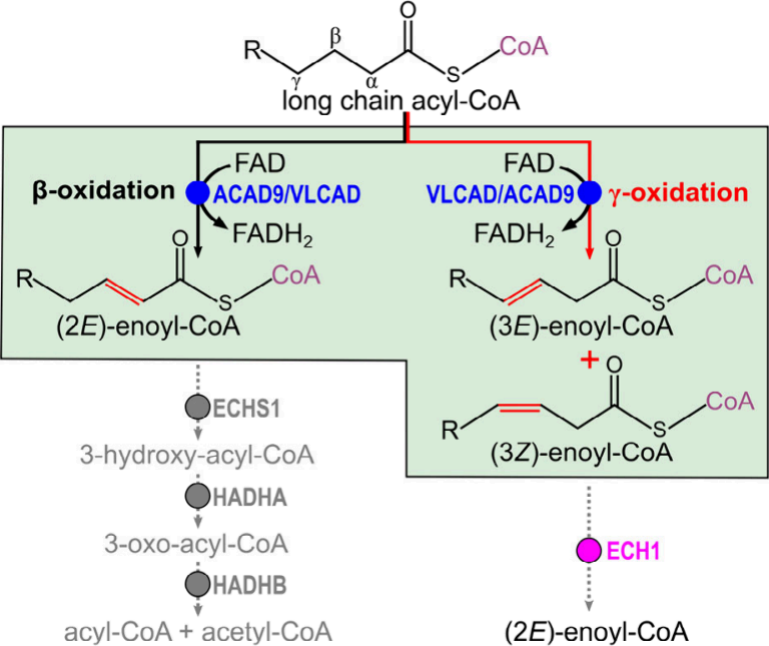
Alternative γ-oxidation pathway. In canonical LC-FAO (black line) ACAD catalyze the α,β-dehydrogenation of acyl-CoA to produce (2*E*)-enoyl-CoA, which proceeds through FAO cycle through other enzymatic steps (ECHS1 for enoyl-CoA hydratase, HADHA and HADHB for hydroxyacyl-CoA dehydrogenase alpha and beta subunits, respectively). In γ-oxidation (red line), ACAD catalyze the formation of (3*E*)-enoyl-CoA and (3*Z*)-enoyl-CoA products. These noncanonical intermediates require isomerization by enoyl-CoA isomerases (e.g., ECH1) to convert them into (2*E*)-enoyl-CoA, enabling entry into the canonical FAO cycle.

Numerous mutations have been identified in both ACAD9 and VLCAD genes, which are linked to severe mitochondrial diseases.^26,27^ However, the key determinants underlying the molecular mechanisms of FAO deficiencies remain largely unclear. This study provides a foundation for future research on disease-associated ACAD mutants, which could help determine whether the alternative β,γ-dehydrogenation pathway functions as an adaptive regulatory mechanism within LC FAO and plays a role in pathological processes.

A deeper understanding of this reaction will also require investigating the substrate preference of β,γ-dehydrogenation. While our study focused on C16:0-CoA and C16:1-CoA, testing additional substrates, such as C14- and C18-CoA, will be important to determine whether γ-oxidation is a general feature of VLCAD and ACAD9 or specific to mid-chain long-chain acyl-CoAs. Furthermore, extending this investigation to other key mitochondrial ACADs, such as MCAD and SCAD (Short-Chain Acyl-CoA Dehydrogenase), could reveal whether β,γ-dehydrogenation is restricted to long-chain fatty acid oxidation or represents a broader enzymatic mechanism across different substrate classes. Future studies addressing these aspects will provide further insights into the biochemical and metabolic significance of γ-oxidation in mitochondrial FAO.

## Supporting Information Available

Additional experimental details, including materials and methods. FAD cofactor characterization of ACAD9 and VLCAD. ESI-MS and NMR characterization of all the substrates before and after incubation with ACAD9 or VLCAD. Quantification of the relative percentages of stereoisomers (PDF)
