# Supplementary material for: Hidden β-γ Dehydrogenation Products in Long-Chain Fatty Acid Oxidation Unveiled by NMR: Implications on Lipid Metabolism
Source: ACS Bio Med Chem Au. 2025 Mar 15;5(2):262–7. doi: 10.1021/acsbiomedchemau.4c00140 (PMC12006827; doi:10.1021/acsbiomedchemau.4c00140)
Supplement: Supplementary file 1 — bg4c00140_si_001.pdf [file bg4c00140_si_001.pdf]

Supporting information for

Hidden β-γ dehydrogenation products in long-chain fatty acid oxidation unveiled by NMR: implications on lipid metabolism

Simone Fabbian[#,§], Beatrice Masciovecchio[#], Elisabetta Schievano[#] and Gabriele Giachin[#]

[#] Department of Chemical Sciences, University of Padua, via F. Marzolo 1, 35131, Padova, Italy;
[§] Department of Pharmaceutical and Pharmacological Sciences, University of Padua, via F. Marzolo 5, 35131, Padova, Italy.

* Corresponding Author: Gabriele Giachin, gabriele.giachin@unipd.it

Table of Contents **page**

Material and Methods 2
Figure S1: *FAD Cofactor Characterization in ACAD9 and VLCAD.* 5
Figure S2: *ETF reduction fluorescence assays of ACAD9 and VLCAD with C16:0-CoA substrate.* 6
Figure S3: *ESI-MS characterization of C16:0-CoA substrate.* 7
Figure S4: ^{1}H-^{1}H TOCSY *characterization of C16:0-CoA substrate.* 8
Figure S5: *ESI-MS characterization of C16:0-CoA substrate in presence of ACAD9.* 9
Figure S6: *ESI-MS characterization of C16:0-CoA substrate in presence of VLCAD.* 10
Figure S7: *HSQC spectrum of ^{13}C C16:0-CoA substrate after incubation with VLCAD.* 11
Figure S8: *Relative percentages of β- and γ-oxidized products and (3E)- to (3Z)-stereoisomers.* 12
Figure S9: *ESI-MS characterization of (9Z)-C16:1-CoA substrate.* 13
Figure S10: ^{1}H-^{1}H TOCSY *characterization of (9Z)-C16:1-CoA substrate*. 14
Figure S11: *ESI-MS characterization of (9Z)-C16:1-CoA substrate in presence of ACAD9.* 15
Figure S12: ^{1}H-^{1}H TOCSY *characterization of (9Z)-C16:1-CoA substrate in presence of ACAD9*. 16
Figure S13: *Structures of (2,9Z)-Hexadecadienoyl-CoA and (3,9Z)-Hexadecadienoyl-CoA.* 17
Figure S14: *^{1}H-NMR of palmitoyl-CoA (C16:0-CoA).* 18
Figure S15: *^{1}H-NMR of palmitoleoyl-CoA ((9Z)-C16:1-CoA).* 19
Figure S16. *Phylogenetic analysis of human ACADs and enoyl-CoA isomerases.* 20

Table S1: *m/z values of the C16:0-CoA mass spectrum.* 21
Table S2: *m/z values of C16:0-CoA in presence of ACAD9.* 22
Table S3: *m/z values of C16:0-CoA in presence of VLCAD.* 23
Table S4: *m/z values of the (9Z)-C16:1-CoA mass spectrum.* 24
Table S5: *m/z values of (9Z)-C16:1-CoA in presence of ACAD9.* 25
Table S6. *^{1}H and ^{13}C NMR assignments for C16:0-CoA.* 26
Table S7. *^{1}H NMR assignments for (9Z)-C16:1-CoA.* 27
Table S8. *^{1}H and ^{13}C NMR assignments for C16:0-CoA after incubation with ACAD9 or VLCAD.* 28
Table S9. *^{1}H NMR assignments for (9Z)-C16:1-CoA after incubation with ACAD9.* 29

Supporting Information References. 30

Material and Methods

1. Reagents, main instrumentation and software

Enzyme substrates, including palmitoyl-CoA (hexadecanoyl-CoA or C16:0-CoA, cat. no. P9716), uniformly $^{13}C_{16}$-labeled palmitoyl-CoA (cat. no. 655716), and palmitoleoyl-CoA -also denoted as (9*Z*)-hexadec-9-enoyl-CoA or (9*Z*)-C16:1-CoA-cat. no. P6775), were purchased from Merck as lithium salt adducts. For protein purification, standard reagents were used, including sodium phosphate (Na_2HPO_4), sodium chloride (NaCl), EDTA disodium salt, imidazole, DL-dithiothreitol (DTT), Triton X-100, glycerol, and Deoxyribonuclease I (DNase I) from bovine pancreas, all obtained from Merck. Magnesium chloride ($MgCl_2$) was sourced from Alfa Aesar, and EDTA-free protease inhibitor cocktail tablets were purchased from Roche. Deuterated water (D_2O, 99.90%) was supplied by Eurisotop. Bacterial harvesting was performed using an Allegra centrifuge (Beckman Coulter). FPLC purifications were conducted on an ÄKTA purifier system, utilizing a 5 mL HisTrap FF crude column and a Superdex 200 Increase 10/300 column, both provided by Cytiva. Protein UV spectra were recorded using a Shimadzu UV-2501PC spectrophotometer equipped with a double monochromator and both a 50W halogen lamp and a deuterium (D2) lamp. Mass spectrometry (MS) experiments were performed via ESI-MS on a QTof Xevo G2S mass spectrometer (Waters, Manchester, UK), with positive mode used for palmitoyl-CoA and negative mode for palmitoleoyl-CoA. One-dimensional (1D) and two-dimensional (2D) nuclear magnetic resonance (NMR) spectra were acquired at 25°C on a Bruker AVANCE NEO 600 MHz spectrometer, equipped with a 5 mm Prodigy TCI cryogenic probe. All NMR spectra were processed using Bruker Topspin 4.0.6 (Bruker BioSpin GmbH, Rheinstetten, Germany). Two-dimensional spectra were visualized and analyzed using NMRFAM-Sparky. First-order ^{1}H-NMR splitting patterns were assigned as singlet (s), doublet (d), or triplet (t), while unresolved splitting patterns were classified as multiplet (m). The molecular structures were drawn using ChemDraw (version 23.1.1), while protein structures were rendered using Chimera X (version 1.8)[1]. The structural model of ACAD9 homodimer in complex with ETF was generated using Alphafold Server[2] (https://alphafoldserver.com/); input sequences for ACAD9 was extracted from PDB ID 8PHF[3], for ETF-alpha subunit from Addgene plasmid #85110 and for ETF-beta subunit from Addgene plasmid #85111). Ligands such as FAD and palmitic acid were also inserted using Alphafold. All the plots were generated using OrginPro 2022 (version 9.9.0.220).

2. Experimental Procedures

<u>*2.1 Expression and purification of human VLCAD and ACAD9*</u>

The expression vector pET-21d(+) encoding for human ACAD9 (residues 38-621, UniProt id Q9H845) was kindly gifted by Dr Montserrat Soler-Lopez from the European Synchrotron Radiation Facility (ESRF), Grenoble, France and used in our previous studies[3-4]. Human VLCAD (residues 75-655, UniProt id P49748)-encoding plasmid was a gift from Nicola Burgess-Brown (Addgene plasmid #38838). ACAD9 and VLCAD expression and purification were carried out according to previously established protocol[3-5]. Slight modifications were introduced to the purification process to make the proteins suitable for NMR studies by avoiding the use of buffers that interfere with NMR analysis. Specifically, bacterial pellets were resuspended in 50 mL of ice-cold lysis buffer (100 mM Na_2HPO_4, 300 mM NaCl, 1 mM DTT, 0.25 mM EDTA, 0.2% v/v Triton X-100, pH 8) supplemented with protease inhibitor cocktail, DNase I, and 1 mg/mL $MgCl_2$. Bacterial lysis was achieved using a FrenchPress (Thermofisher) and the lysate was clarified by centrifugation at 12,000 g for 1 hour at 4°C. The cleared lysate was loaded onto a 5 mL-HisTrap™ FF crude column, equilibrated with binding buffer (100 mM Na_2HPO_4, 300 mM NaCl, pH 8). Protein elution was performed using a linear imidazole gradient (0-100% imidazole over 25 mL). Fractions containing the target proteins, identified by their characteristic yellow colour due to the presence of the FAD^+ prosthetic group, were collected and concentrated using ultrafiltration devices (Sartorius, Vivaspin 10 kDa MWCO). The concentrated proteins were further purified by size-exclusion chromatography (SEC) using HiLoad 200 16/600 or a Superdex 200 Increase 10/300 (Cytiva) column equilibrated with NMR buffer (20 mM Na_2HPO_4, 50 mM NaCl, pH 8). Elution was monitored at 280 nm to detect protein content and 450 nm to confirm the presence of the FAD cofactor bound to the enzymes. Fractions containing ACAD9 or VLCAD were pooled, concentrated to a final concentration of 0.6 mM, and

stored at 4°C for subsequent MS and NMR experiments. The concentration of ACAD9 and VLCAD was estimated using UV absorption spectra. Approximately 100-150 µL of the protein sample was transferred to a 1 cm path-length, low-volume quartz cuvette. UV spectra were recorded from 350 nm to 230 nm. Protein concentration was determined using the Lambert-Beer law at 280 nm, with molar extinction coefficients (ε280) of 34,840 M^{-1} cm^{-1} for ACAD9 and 42,400 M^{-1} cm^{-1} for VLCAD, respectively. The calculation provided a reliable estimate of protein concentration based on the absorbance at 280 nm. The estimation of FAD content in ACAD9 and VLCAD was confirmed formed using UVvis spectrophotometer (UV-2401PC, Shimadzu) at 10 µM protein concentration in NMR buffer. Absorbance spectra were recorded with 1 nm steps within a wavelength range from 230 nm to 600 nm using a 10 mm light path quartz cuvette.

2.2 Acyl-CoA dehydrogenase (ACAD) activity assay

We used the ETF fluorescence reduction assay[6] for sensitive determination of ACAD9 and VLCAD activities, since ETF is the natural electron acceptor for the ACAD enzymes[7]. The plasmids of the human electron transfer flavoprotein ETF-alpha and ETF-beta subunits were a gift from Pal Falnes (pF710-pETDuet-1-Hs-delta19-ETFalpha-noHis and pF709-pET28a-Hs-FL-ETFbeta-NHis, Addgene plasmids #85110 and #85111, respectively). Purification of the recombinant ETF alpha/beta heterodimer was carried out as previously described[4, 8]. Briefly, *E. coli* C43(DE3) cells (Lucigen) were co-transformed with the plasmids, induced with 500 µM IPTG and harvested 12 hours after induction at 37 °C. Cells were lysed in lysis buffer containing 25 mM HEPES (Thermofisher, cat.no. 10521241), 250 mM NaCl, 20 mM imidazole, 0.2% Tween-20 (Merck, T2700) at pH 7.8 and supplemented with protease inhibitor cocktails (Merck) and DNase I (Merck). Cleared lysates were loaded onto a 5-mL HisTrap column (Cytiva) equilibrated in the binding buffer (25 mM HEPES, 250 mM NaCl, 20 mM imidazole, pH 7.8). Bound proteins were eluted with elution buffer (25 mM HEPES, 250 mM NaCl, 500 mM imidazole, pH 7.8). Elution fractions containing ETF heterodimer were pooled, dialysed against 50 mM Tris (Fluka, cat. no. 93352) at pH 8.0 and purified on a Superdex 200 Increase 10/300 SEC column (Cytiva). Purified samples were stored at -80 °C after the addition of 0.5% glycerol.

Anaerobic ETF fluorescence reduction assays were done as previously described[4, 6], with reaction volumes of 150 µL in standard black microplates with clear plastic bottoms (Greiner Bio-One #655090). Reactions were measured in a plate reader Infinite M200 PRO (TECAN) set to 32 °C, using $Ex340_{nm}/Em490_{nm}$. Approximately 30 data points were collected for each sample over a 50-70 seconds measurement window. Glucose oxidase (Sigma, G2133, 20 U/mL final concentration) and catalase (Sigma, C30, 0.021 mg/mL final concentration) were added and fluorescence was zeroed. Then, enzyme sample (3.75 µg, 0.0025 mg/ml, recombinant ACAD9 or VLCAD) and human ETF (85 µg, 9 µM final concentration) in buffer 50 mM Tris-HCl, 0.5% w/v glucose, pH 8.0 were added. Background fluorescence was recorded for 50-70 seconds. The reaction was initiated by addition of 0.03 mM final concentration of palmitoyl-CoA (C16:0-CoA) substrate (Sigma P6775) and immediately read for 50-70 s. Each experiment was performed in 5-10 replicates. Slopes and Y-intercepts were calculated using OriginPro 2022 (version 9.9.0.220) and used in the Equation 1 given below to calculate specific activity in mU following a method previously described[6].

Equation 1: $$\frac{(\left(\frac{slope}{Y}-int\right)*nmoles\ ETFFAD*(60\ s/0.91))}{mg\ sample}$$

2.3 MS and NMR samples preparation and measurement

C16:0-CoA (both $^{13}C_{16}$-labeled and unlabeled) and (9*Z*)-C16:1-CoA were initially solubilized in D_2O at a final concentration of 5 mM and stored at −20°C.

Samples for MS analysis were prepared by mixing 1 µL of 5 mM substrate solution, 0.3 µL of NMR buffer, and 48.7 µL of Milli-Q water, resulting in a total volume of 50 µL with a final substrate concentration of 100 µM. To assess the dehydrogenation products, purified ACAD9 or VLCAD was added to the substrates (0.3 µL of a 0.6 mM enzyme solution was added, giving a final enzyme concentration of 3.6 µM) under the same volume proportions as for the C16:0-CoA or (9*Z*)-C16:1-CoA samples. The mixture was incubated overnight at room temperature (25°C). After incubation, the samples were dissolved in methanol (1:1) and directly injected into the mass spectrometer for analysis.

For both the substrate (acyl-CoA alone) and the mixture samples (acyl-CoA plus enzyme), NMR tubes were prepared with a final concentration of 70 μM for either C16:0-CoA or (9*Z*)-C16:1-CoA in D_2O at pH 7. ACAD9 or VLCAD were added to the mixture tubes at a final concentration of 3.6 μM. 1D ^{1}H-NMR experiments were performed using the Bruker "zgesgp" excitation sculpting pulse sequence to suppress the residual water signal. Data were collected with 128 scans, 32K data points and an acquisition time of 6 minutes. 2D ^{1}H-^{1}H TOCSY experiments were conducted using the Bruker "mlevphpr.2" pulse sequence, with 2K × 720 data points, 100 increments in the indirect dimension, a recovery delay of 1.0 seconds, and a spin-lock mixing time of 70 ms. The total acquisition time for the 2D TOCSY experiments was 25 hours. For ^{1}H-^{13}C HSQC experiments, uniformly $^{13}C_{16}$-labeled palmitoyl-CoA samples were used. The Bruker "hsqcetgpsisp.2" pulse sequence was employed with 2K × 256 data points, 128 increments in the indirect dimension, and a recovery delay of 1.5 seconds. The total acquisition time for the HSQC experiments was 9 hours and 50 minutes. ^{1}H and ^{13}C assignments of starting substrates, C16:0-CoA, (9*Z*)-C16:1-CoA, were carried-oud following previous reports[9-10] and Metabolome Database (HMDB, https://hmdb.ca/)[11]. The relative percentages of β-oxidized product and γ-oxidized products (both E and Z isomers) were assessed based on the intensity of allylic methylene cross-peak. These were determined from the 1H-13C HSQC spectra of palmitoyl-CoA in the presence of either ACAD9 or VLCAD, using NMRFAM-Sparky for data analysis. The same procedure was used to quantify the ratio between (3E)-hexadecenoyl-CoA and (3Z)-hexadecenoyl-CoA.

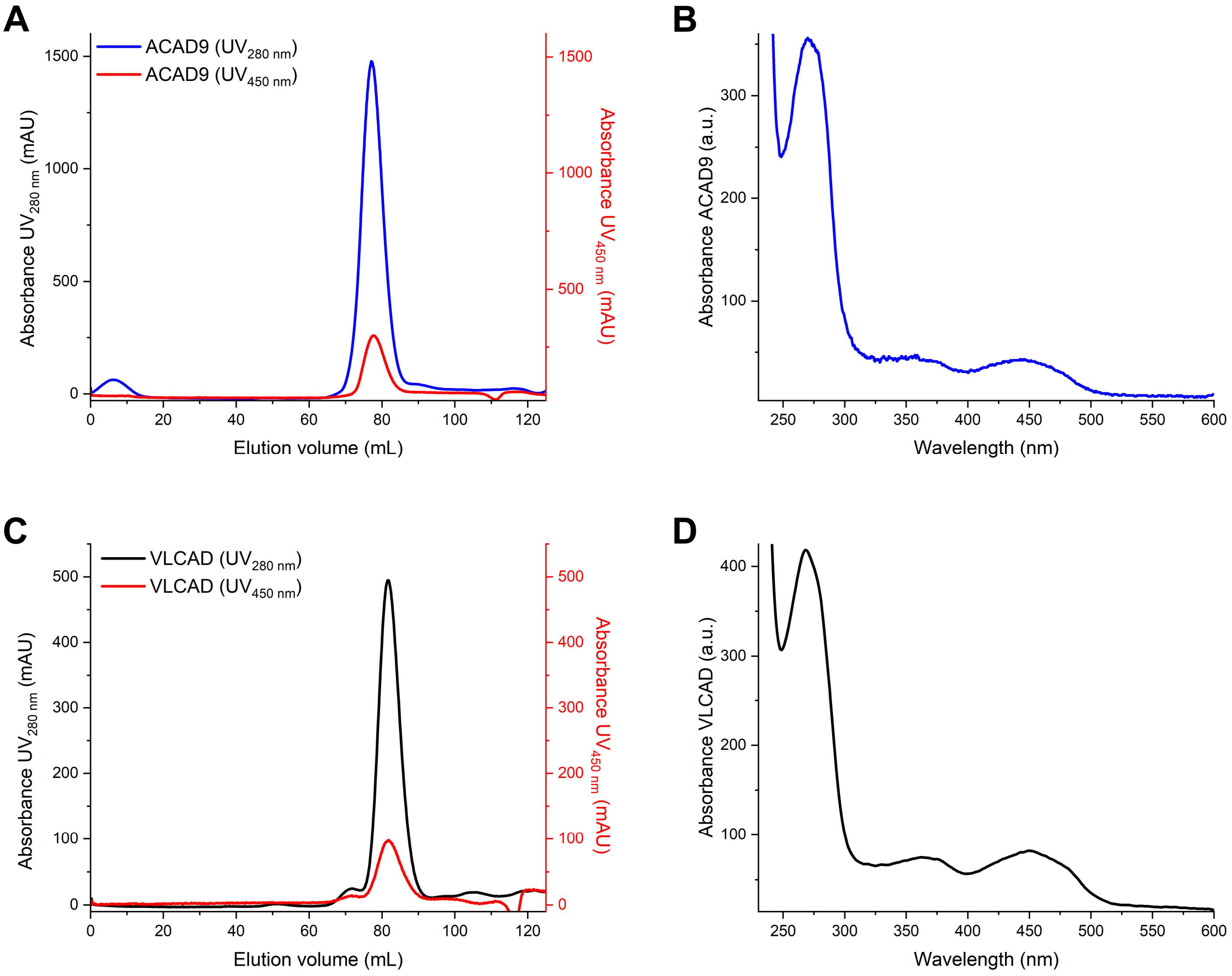

Figure S1. *Characterization of ACAD9 and VLCAD enzyme preparations confirming the presence of the FAD cofactor*. **(A, C)** Size exclusion chromatography purification profiles of ACAD9 (A) and VLCAD (C) using a HiLoad 16/600 Superdex 200 column. Elution was monitored at 280 nm (blue/black traces) to detect protein content (tryptophan) and at 450 nm (red traces) to assess the presence of the FAD cofactor, which absorbs in this region. Both ACAD9 and VLCAD elute as single peaks, with a corresponding UV450 signal indicating FAD binding. **(B, D)** UV-Vis absorption spectra of ACAD9 (B) and VLCAD (D), showing characteristic peaks at 280 nm and near 370 nm and 450 nm, consistent with the presence of oxidized FAD in both enzymes.

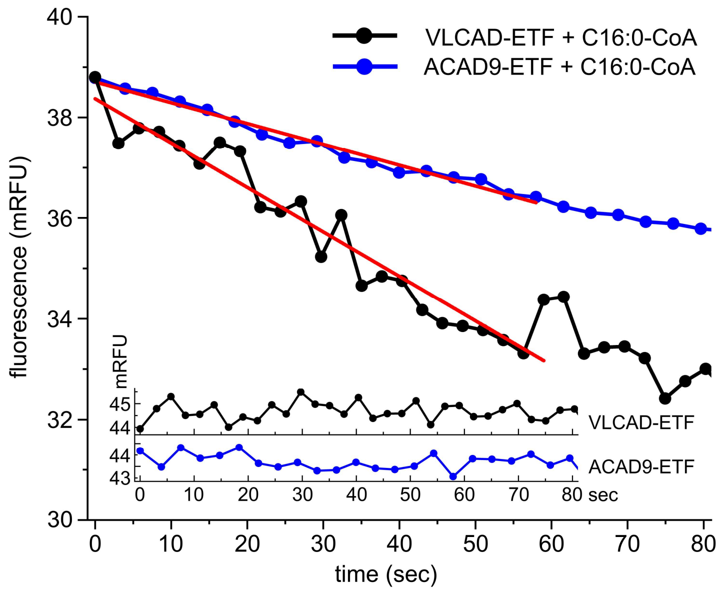

Figure S2. *ETF reduction fluorescence assays of ACAD9 and VLCAD with C16:0-CoA substrate*. Time-course fluorescence traces displaying ETF reduction during the oxidation of C16:0-CoA by VLCAD (black trace) and ACAD9 (blue trace). The observed fluorescence decrease corresponds to the quenching of ETF fluorescence as electrons are transferred from $FADH_2$ of ACAD9 or VLCAD to oxidized ETF. The *inset* shows the control experiment where ACAD-ETF alone does not exhibit significant fluorescence change in the absence of substrate. Fluorescence measurements were recorded in milli relative fluorescence units (mRFU) over 80 seconds, and linear regression fits (red lines) were applied to the data.

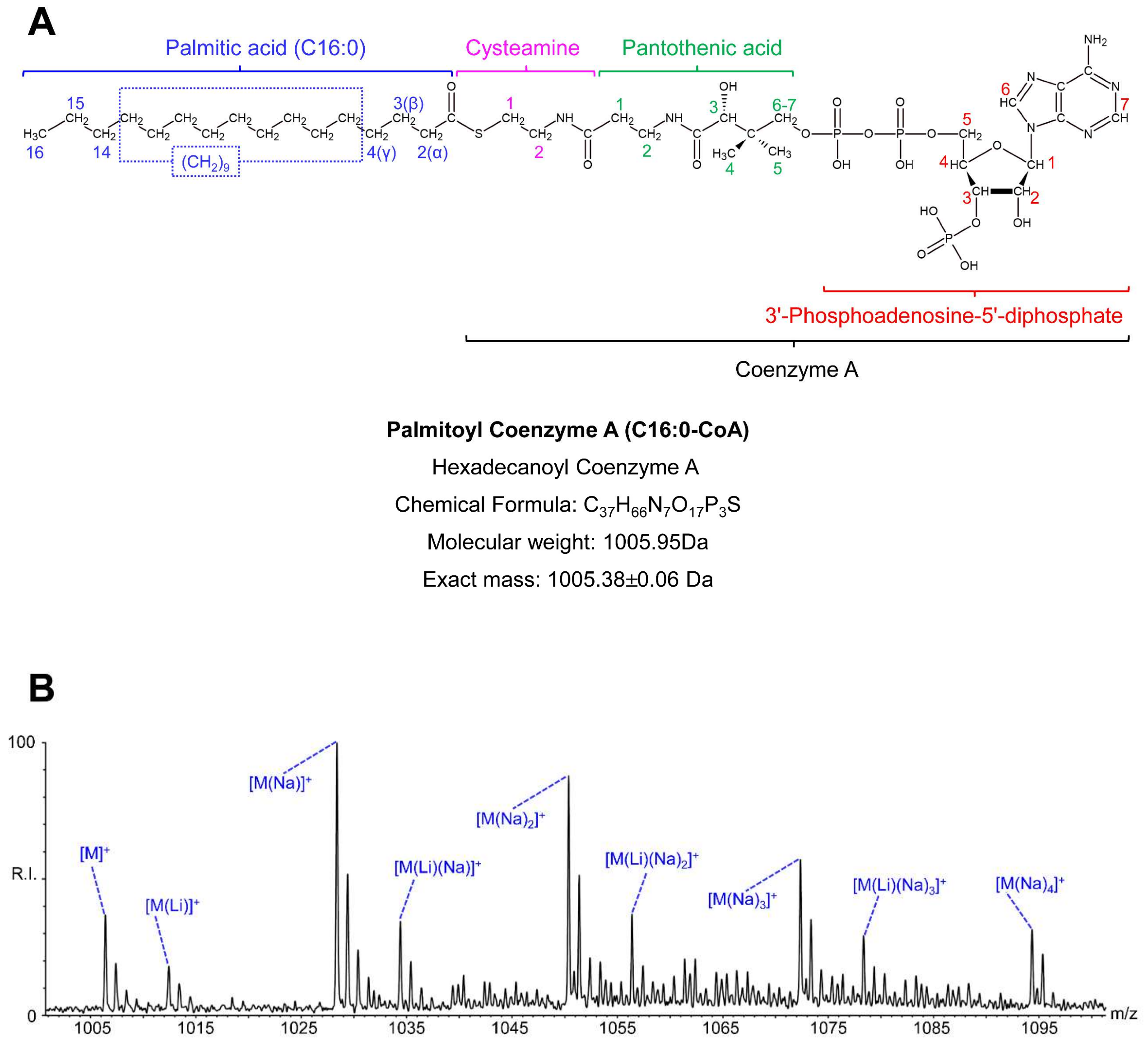

Figure S3. *ESI-MS characterization of C16:0-CoA substrate*. (**A**) Molecular structure of C16:0-CoA. The four blocks forming the substrate structure are indicated with different colors: blue (palmitic acid), purple (cysteamine), green (pantothenic acid) and red (3'-phosphoadenosine-5'-diphosphate). The exact mass was calculated in ESI mass spectrum. (**B**) ESI-MS spectrum of C16:0-CoA in its lithium salt form. The spectrum shows multiple peaks corresponding to the molecular ion [M]+ and its adducts with lithium and sodium. See **Table S1** for details.

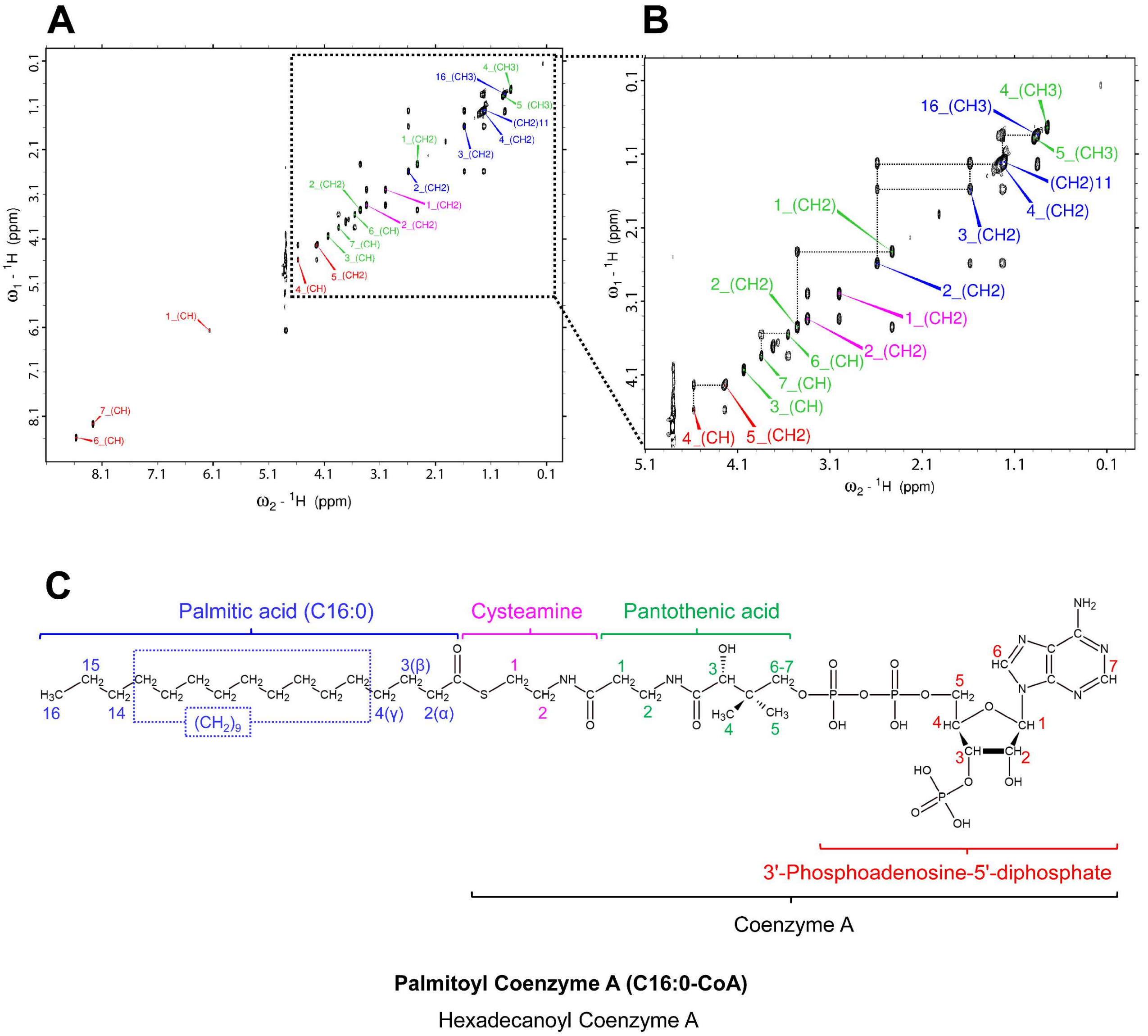

Figure S4. ^1H-^1H TOCSY *characterization of C16:0-CoA substrate.* (**A**) Full ^1H-^1H TOCSY spectrum of C16:0-CoA, with each correlation peak numbered and color-coded to match the regions highlighted in panel C. The spectrum shows the interactions between the different proton groups in the molecule. (**B**) Expanded region of the spectrum, focusing on the key correlations between proton groups from the four major molecular blocks of C16:0-CoA: palmitic acid, cysteamine, pantothenic acid, and 3'-phosphoadenosine-5'-diphosphate. (**C**) Molecular structure of C16:0-CoA, divided into four distinct blocks highlighted by colour: blue (palmitic acid), purple (cysteamine), green (pantothenic acid), and red (3'-phosphoadenosine-5'-diphosphate). These regions correspond to the numbered and color-coded peaks in panels A and B, facilitating clear identification of the individual proton correlations in the TOCSY spectrum.

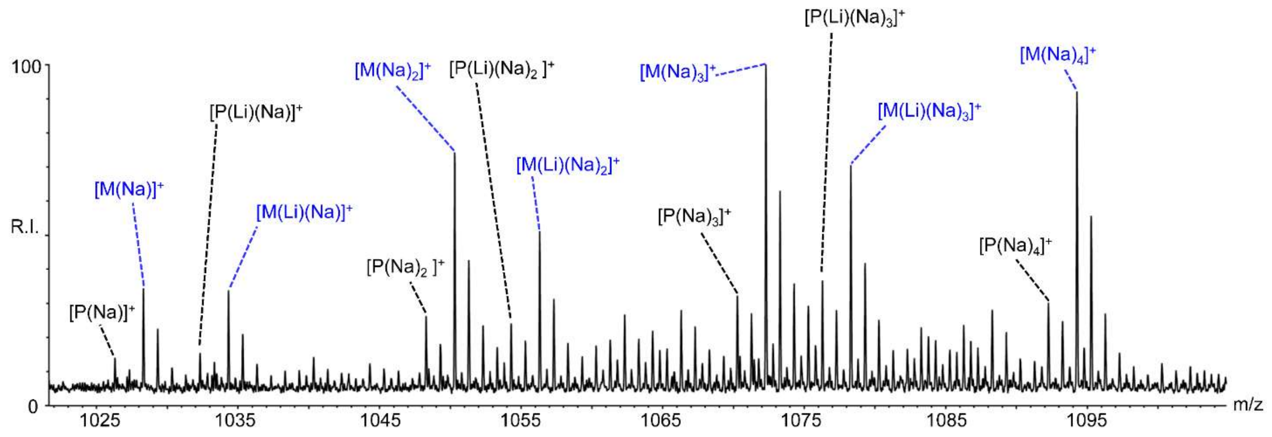

Figure S5. *ESI-MS characterization of C16:0-CoA substrate in presence of* ***ACAD9****.* The spectrum shows multiple peaks corresponding to the molecular ions [M]+ (C16:0-CoA, blue colored), [P]+ (dehydrogenation products (2*E*)-C16:1-CoA, (3*E*)-C16:1-CoA and (3*Z*)-C16:1-CoA, black colored) and their adducts with lithium and sodium. See **Table S2** for detail.

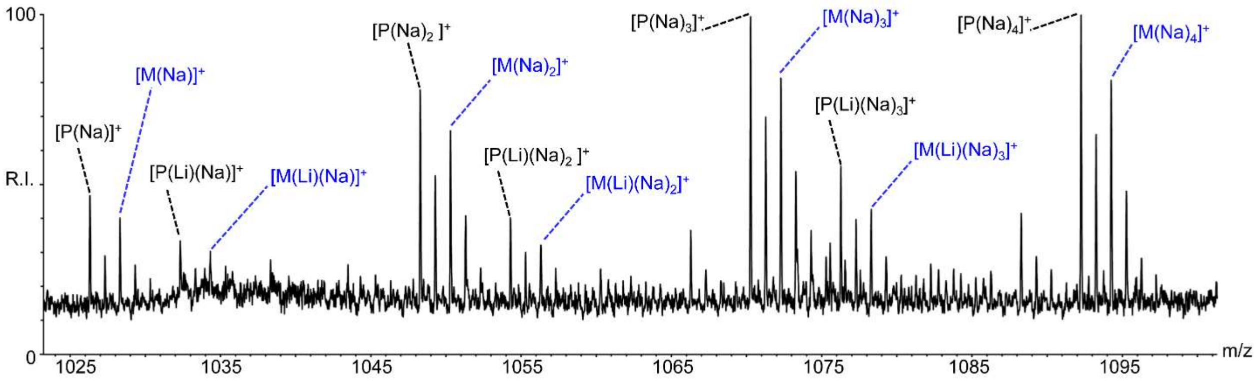

Figure S6. *ESI-MS characterization of C16:0-CoA substrate in presence of **VLCAD**.* The spectrum shows multiple peaks corresponding to the molecular ions [M]+ (C16:0-CoA, blue colored), [P]+ (dehydrogenation products (2*E*)-C16:1-CoA, (3*E*)-C16:1-CoA and (3*Z*)-C16:1-CoA, black colored) and their adducts with lithium and sodium. See **Table S3** for detail.

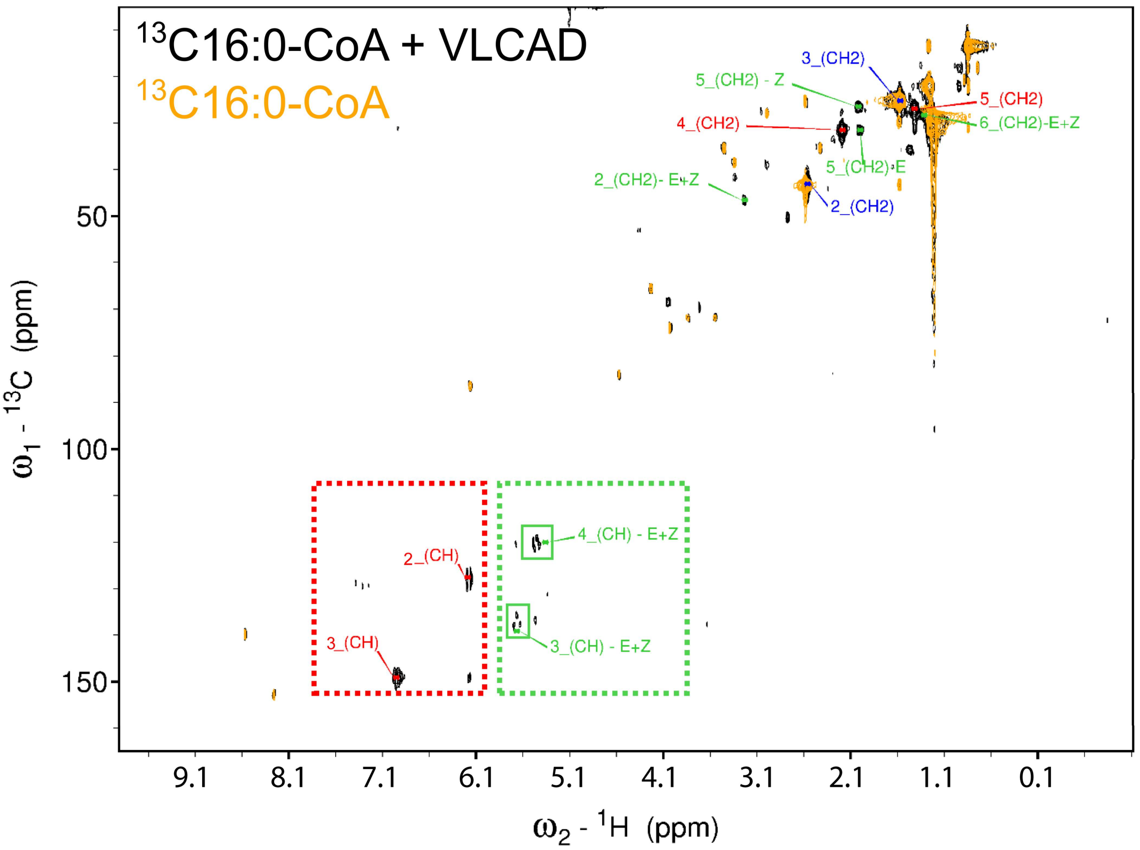

Figure S7. *HSQC spectrum of ^{13}C C16:0-CoA substrate after incubation with* ***VLCAD***, highlighting newly formed desaturation products. The red dotted box indicates the characteristic α,β-dehydrogenation product, (2*E*)-hexadecenoyl-CoA. In the green box: additional signals between 5.4 and 5.7 ppm in the ^{1}H dimension and 120-140 ppm in the ^{13}C dimension suggest a second, novel double bond between carbon β and γ, leading to (3*E*)-hexadecenoyl-CoA and (3*Z*)-hexadecenoyl-CoA.

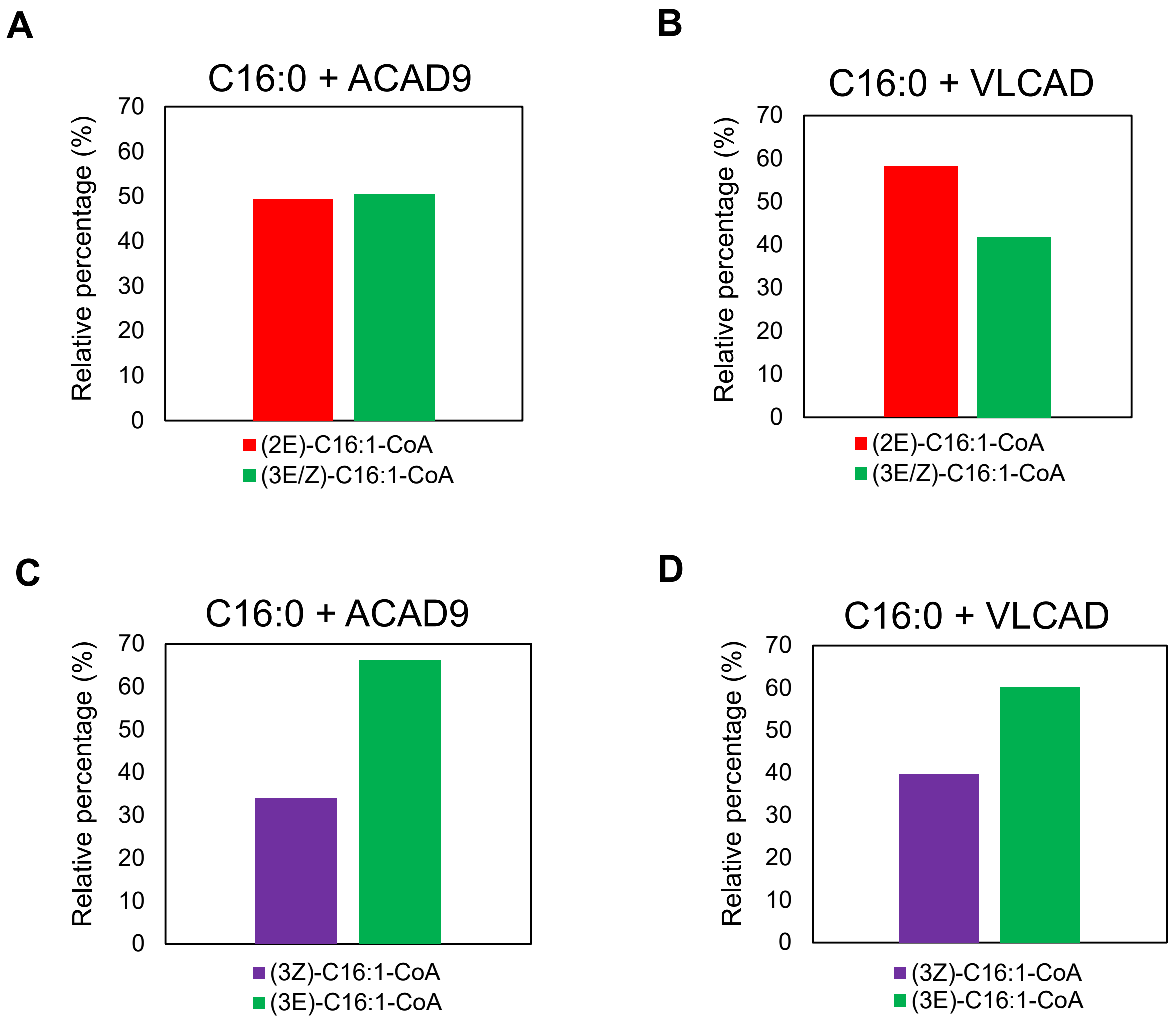

Figure S8. *Relative percentages of β-oxidized and γ-oxidized products, and relative percentages of the E-stereoisomer compared to the Z-stereoisomer based on ^{1}H-^{13}C HSQC analysis.*

Panel **A** and **B**: quantification of the relative percentages between β-oxidized product (2*E*)-hexadecenoyl-CoA [(2*E*)-C16:1-CoA] and γ-oxidized products [(3*E*)-hexadecenoyl-CoA, (3*E*)-C16:1-CoA] and [(3*Z*)-hexadecenoyl-CoA, (3*Z*)-C16:1-CoA] after the dehydrogenation reaction of C16:0-CoA catalyzed by ACAD9 (panel **A**) and VLCAD (panel **B**). The relative amounts of (2*E*)-C16:1-CoA *versus* total γ-oxidized products [(3*E*)-C16:1-CoA + (3*Z*)-C16:1-CoA] obtained with ACAD9 (VLCAD) are 49% *versus* 51%, and 58% *versus* 42%, respectively.

Panel **C** and **D**: Enantiomeric excess of the *E*-stereoisomer compared to the *Z*-stereoisomer. Quantification of the relative percentage of (3*E*)-C16:1-CoA and (3*Z*)-C16:1-CoA stereoisomers after the dehydrogenation reaction of C16:0-CoA with ACAD9 (panel **C**) and VLCAD (panel **D**). The relative amounts of (3*E*)-C16:1-CoA and (3*Z*)-C16:1-CoA obtained with ACAD9 (VLCAD) are 66% (60%) and 34% (40%). These results indicate that both enzymes favor the formation of the (3E) stereoisomer, with ACAD9 displaying a stronger preference.

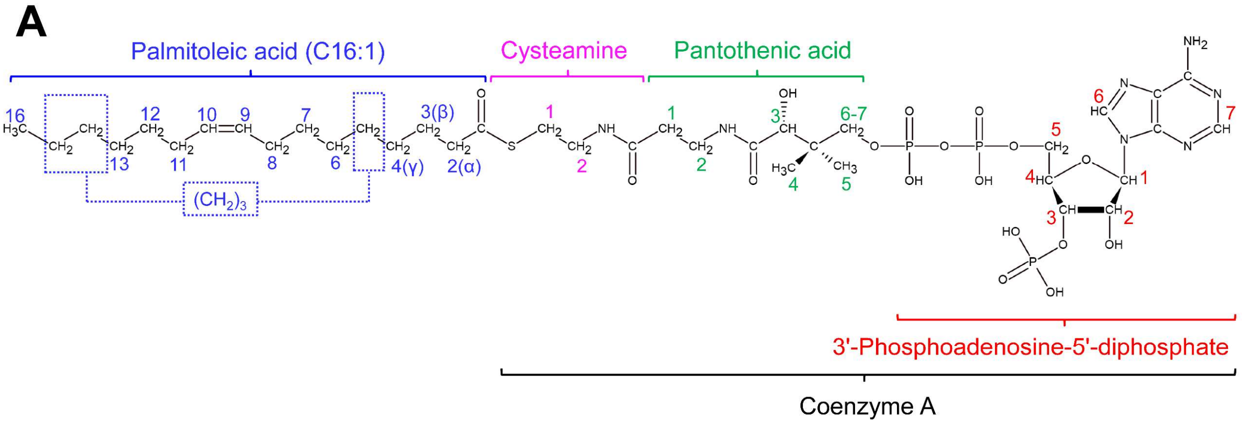

Palmitoleoyl Coenzyme A ((9*Z*)-C16:1-CoA)

(9Z)-Hexadecenoyl Coenzyme A

Chemical Formula: $C_{37}H_{64}N_7O_{17}P_3S$

Molecular weight: 1003.93Da

Exact mass: 1003.36±0.04 Da

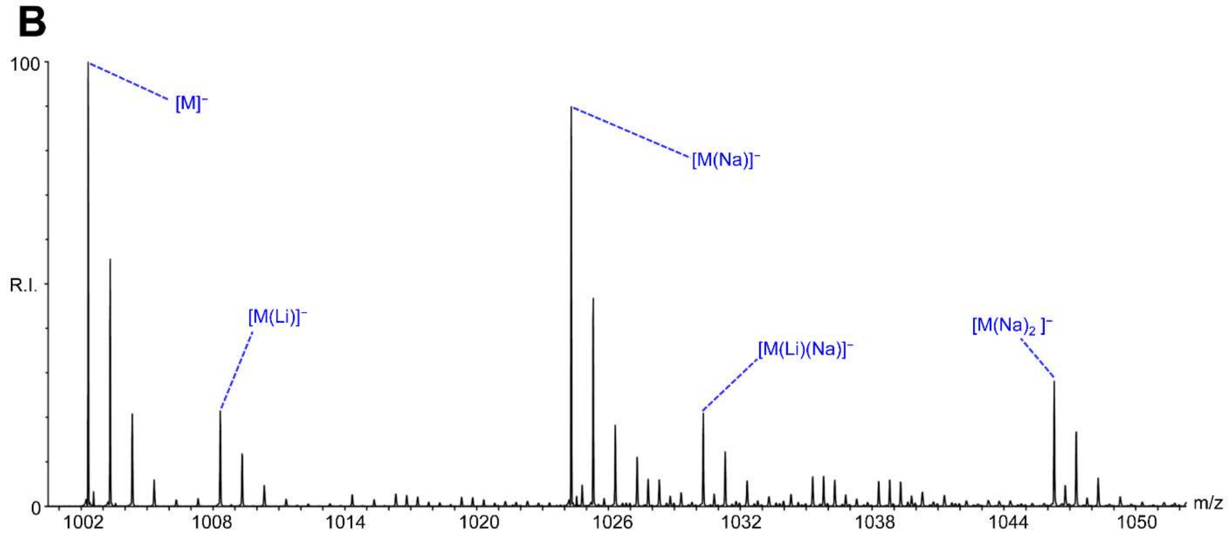

Figure S9: *ESI-MS characterization of (9Z)-C16:1-CoA substrate*. (**A**) Molecular structure of (9*Z*)-C16:1-CoA. The four blocks forming the (9*Z*)-C16:1-CoA structure are indicated with different colours: blue (palmitoleic acid), purple (cysteamine), green (pantothenic acid) and red (3'-phosphoadenosine-5'-diphosphate). The exact mass was calculated by ESI mass spectrum. (**B)** ESI-MS spectrum of (9*Z*)-C16:1-CoA in its lithium salt form. The spectrum shows multiple peaks corresponding to the molecular ion [M]− and its adducts with lithium and sodium. See also **Table S4** for more details.

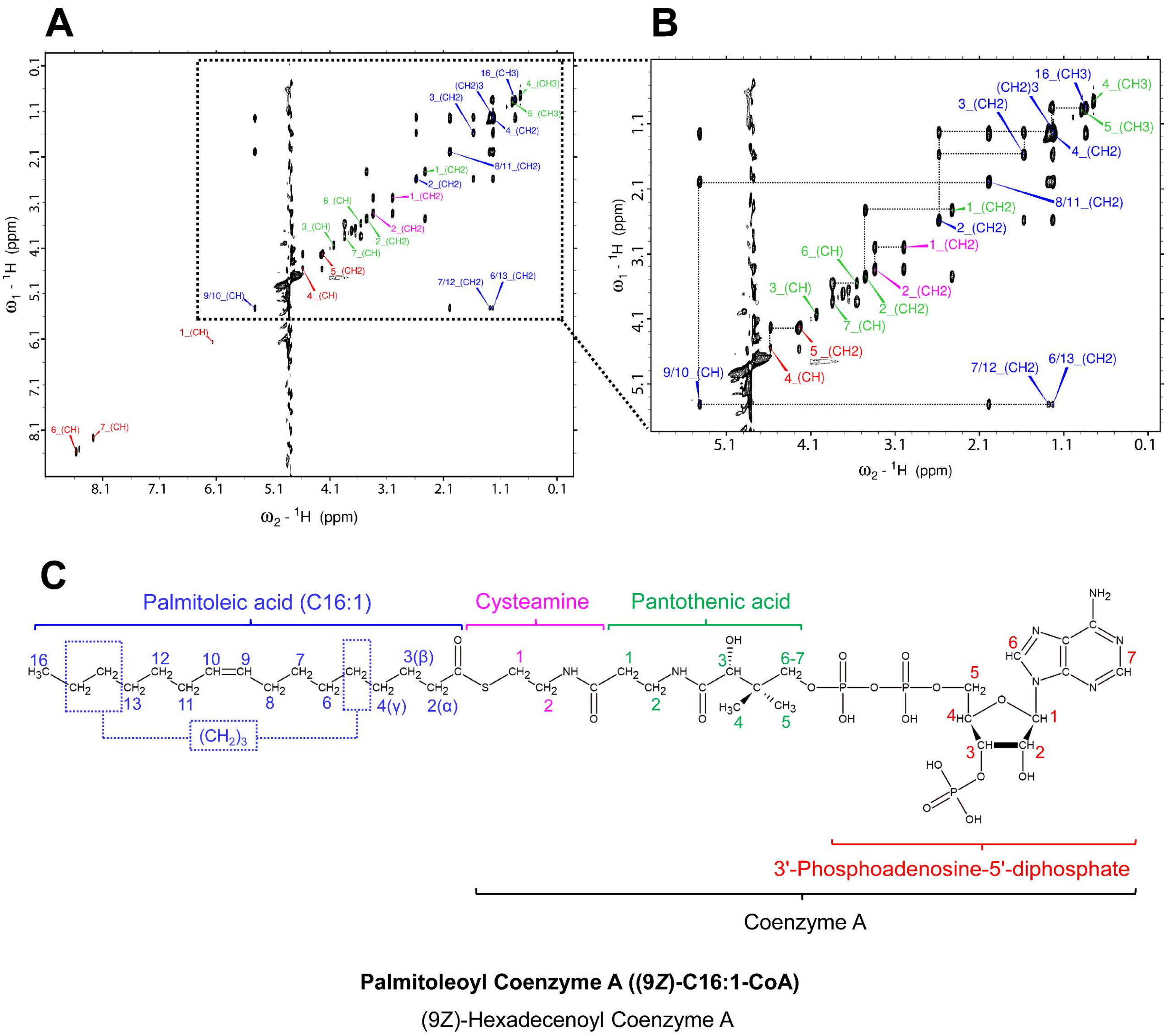

Figure S10. ^{1}H-^{1}H TOCSY *characterization of (9Z)-C16:1-CoA substrate.* (**A**) Full ^{1}H-^{1}H TOCSY spectrum of (9*Z*)-C16:1-CoA, with each correlation peak numbered and color-coded to match the regions highlighted in panel C. The spectrum shows the interactions between the different proton groups in the molecule. (**B**) Expanded region of the spectrum, focusing on the key correlations between proton groups from the four major molecular blocks of (9*Z*)-C16:1-CoA: palmitoleic acid, cysteamine, pantothenic acid, and 3'-phosphoadenosine-5'-diphosphate. (**C**) Molecular structure of (9*Z*)-C16:1-CoA, divided into four distinct blocks highlighted by colour: blue (palmitoleic acid), purple (cysteamine), green (pantothenic acid), and red (3'-phosphoadenosine-5'-diphosphate). These regions correspond to the numbered and coloured peaks in panels A and B, facilitating clear identification of the individual proton correlations in the TOCSY spectrum.

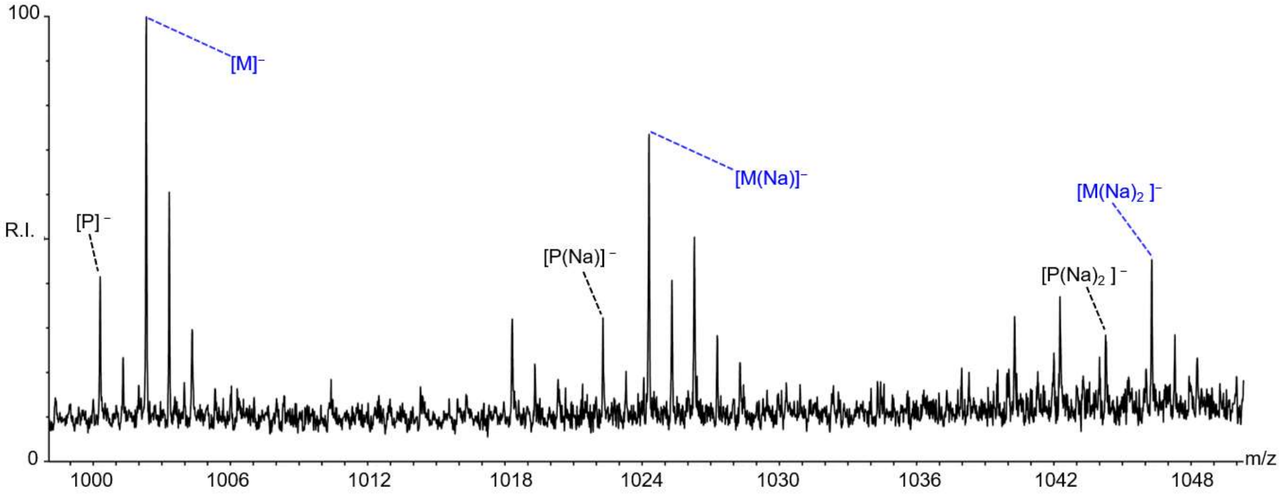

Figure S11. *ESI mass spectrum of (9Z)-C16:1-CoA in presence of* ***ACAD9***. The spectrum shows multiple peaks corresponding to the molecular ions [M]− (C16:0-CoA, blue colored), [P]− (dehydrogenation products (2,9*Z*)- C16:2-CoA and (3,9*Z*)-C16:2-CoA, black colored) and their adducts with lithium and sodium. See **Table S5** for details.

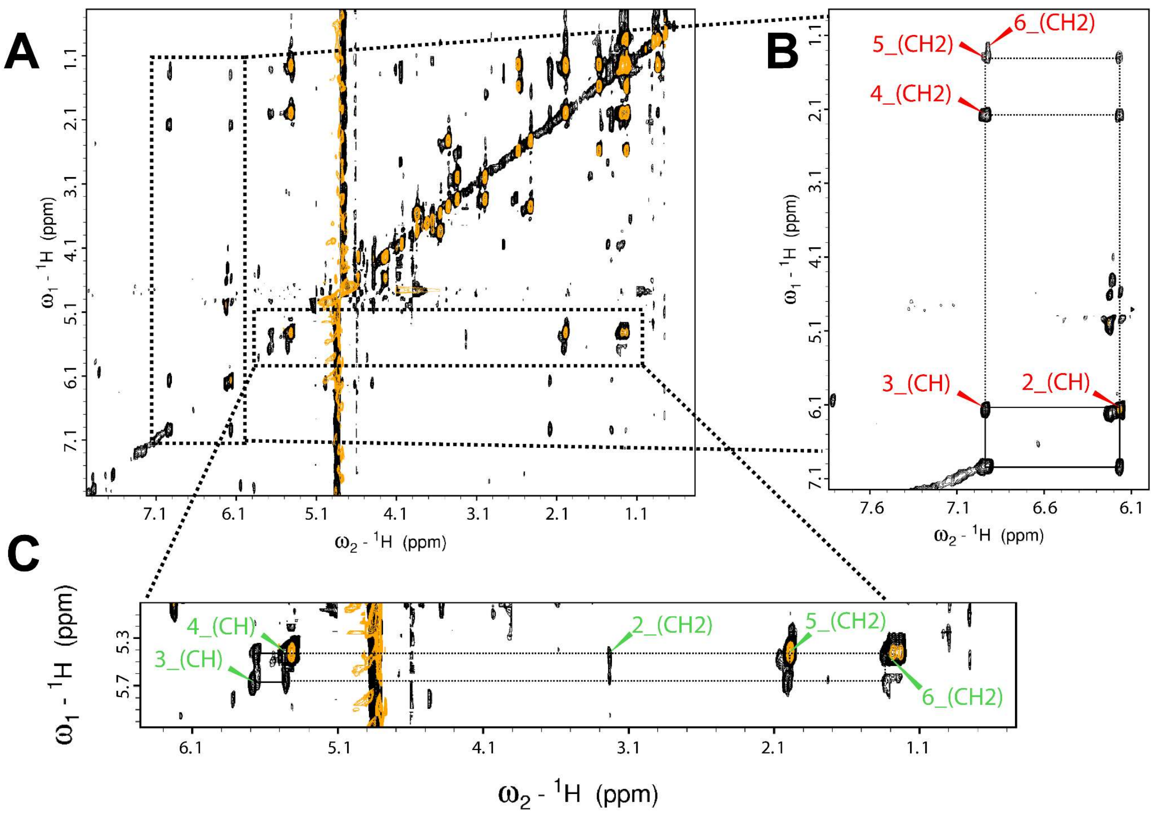

Figure S12. *^{1}H-^{1}H TOCSY characterization of (9Z)-C16:1-CoA substrate in presence of ACAD9*. (**A**) Comparison between the ^{1}H-^{1}H TOCSY spectra of (9*Z*)-C16:1-CoA before (orange spectrum) and after (black spectrum) the incubation with ACAD9. The peaks diagnostic of the formation of the products of dehydrogenation are also indicated in the olefinic region (5-7ppm). The expanded plots show the main correlations between the protons in the acyl chain of (2,9*Z*)-Hexadecadienoyl CoA (**B**) and (3,9*Z*)-Hexadecadienoyl CoA (**C**). Each assignment is indicated with a number and a colour as represented in **Figure S13**.

A

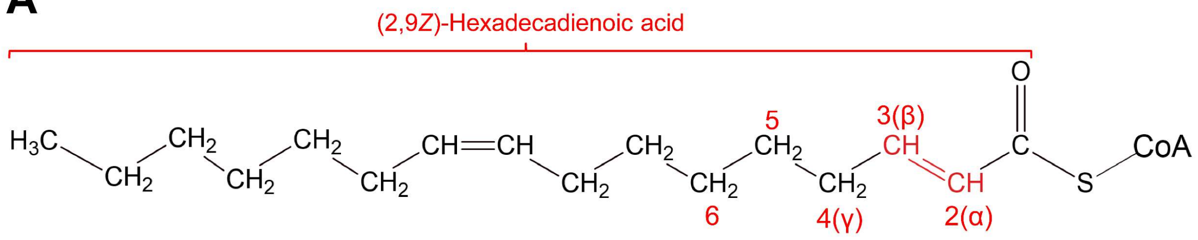

Product 1 ((2,9*Z*)-C16:2-CoA)

(2,9*Z*)-Hexadecadienoyl Coenzyme A

Chemical Formula: $C_{37}H_{62}N_7O_{17}P_3S$

Molecular weight: 1001.91Da

Exact mass: 1001.310±0.004 Da

B

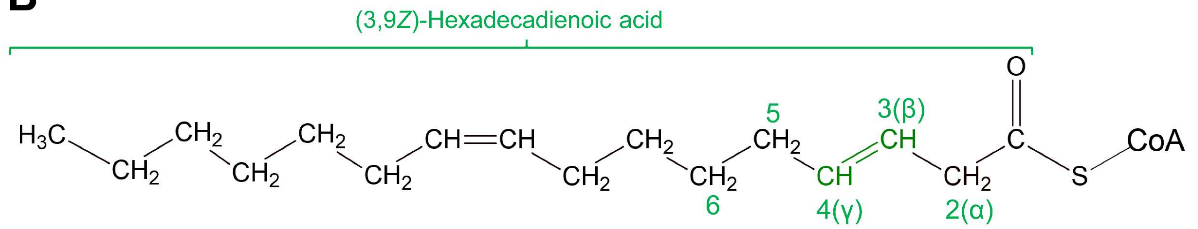

Product 2 ((3,9*Z*)-C16:2-CoA)

(3,9*Z*)-Hexadecadienoyl Coenzyme A

Chemical Formula: $C_{37}H_{62}N_7O_{17}P_3S$

Molecular weight: 1001.91Da

Exact mass: 1001.310±0.004 Da

Figure S13 *Structures of (2,9Z)-Hexadecadienoyl-CoA and (3,9Z)-Hexadecadienoyl-CoA.* (**A**) Product 1 named (2,9*Z*)-Hexadecadienoyl-CoA ((2,9*Z*)-C16:2-CoA), resulting from the α-β dehydrogenation of (9*Z*)-C16:1-CoA substrate by ACAD9. (**B**) Product 2 named (3,9*Z*)-Hexadecadienoyl-CoA ((3,9*Z*)-C16:2-CoA), resulting from the β-γ dehydrogenation of (9*Z*)-C16:1-CoA substrate by ACAD9. The stereochemistry of the double bonds in the products (positions α and β or β and γ along the acyl chain) has not been assigned.

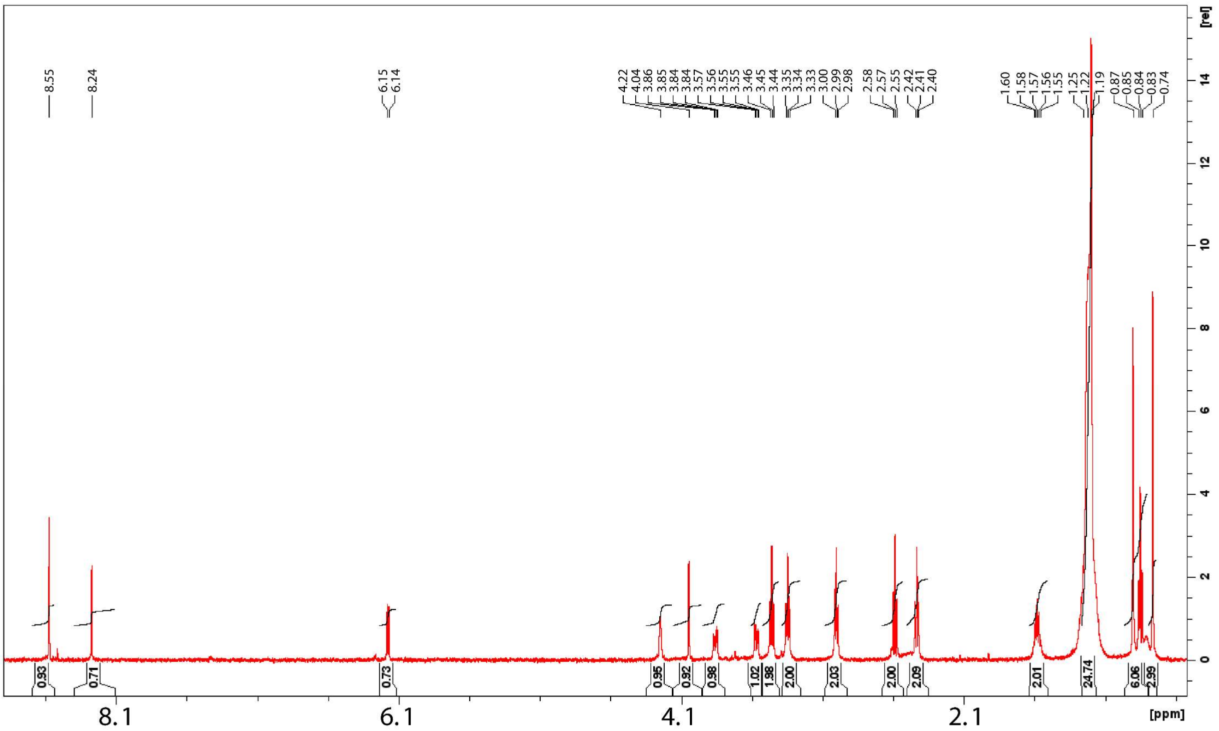

Figure S14: ^{1}H-NMR of palmitoyl-CoA (C16:0-CoA).

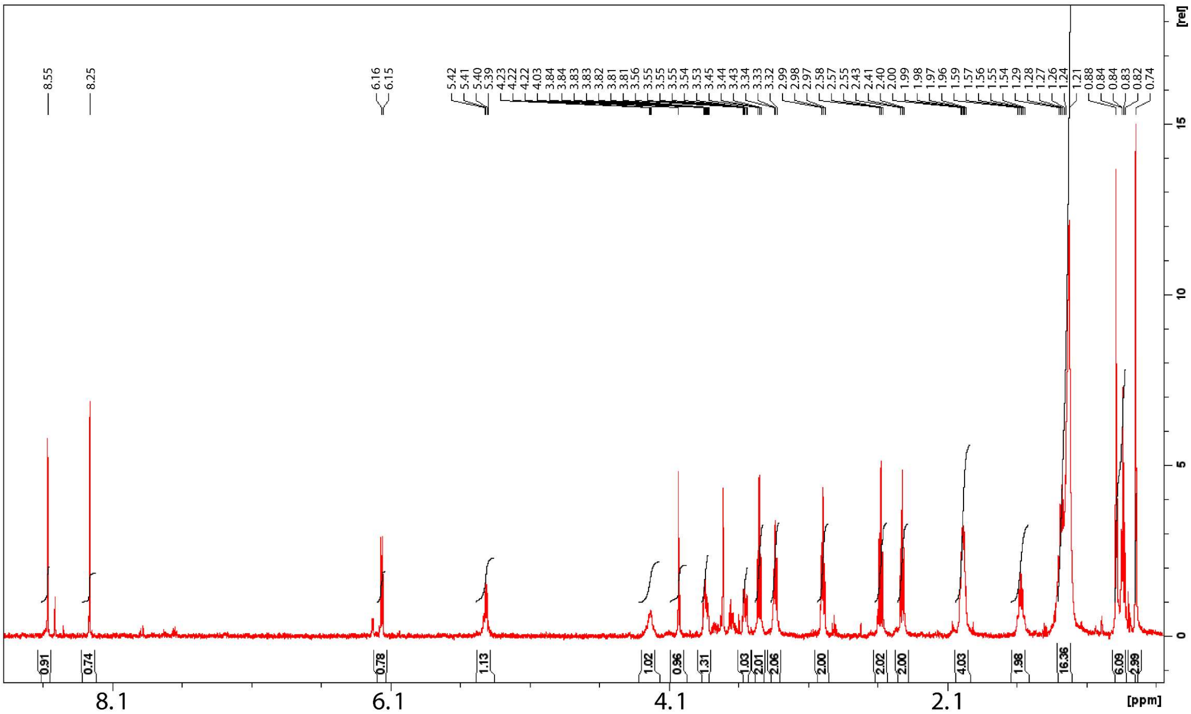

Figure S15: *^{1}H-NMR of palmitoleoyl-CoA* ((9Z)-C16:1-CoA).

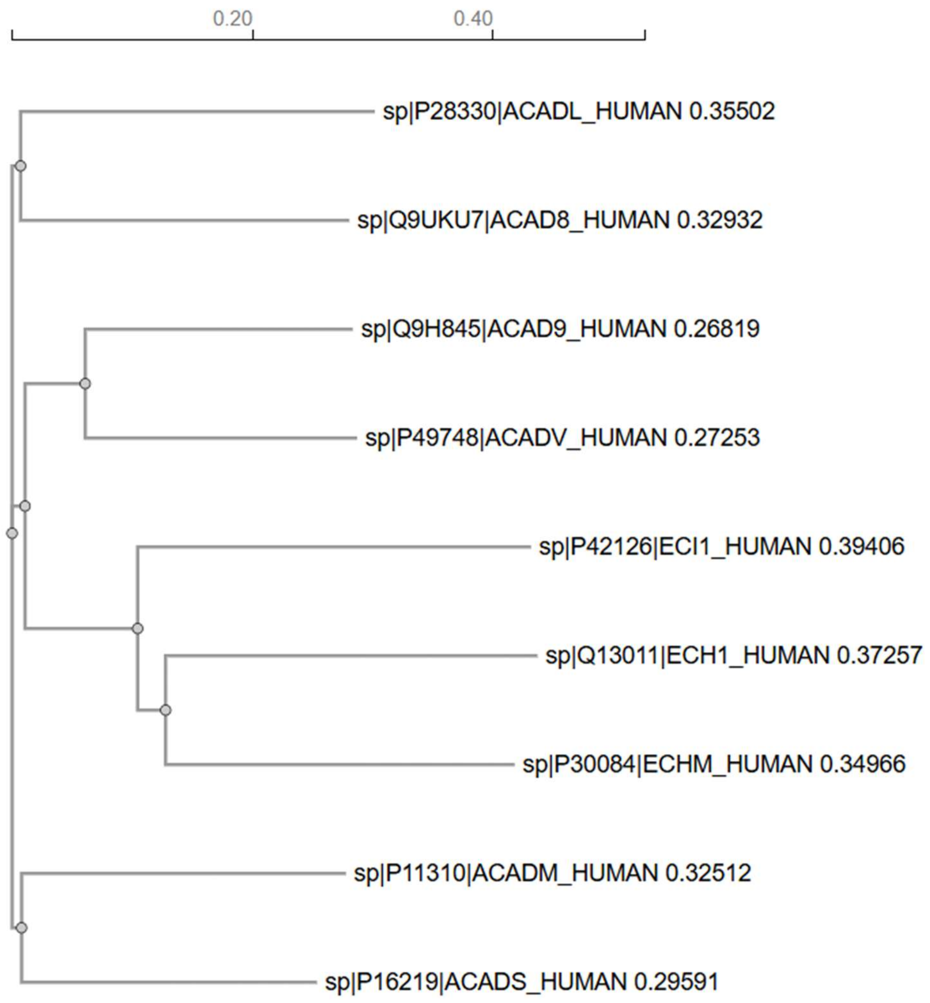

Figure S16. *Phylogenetic analysis of human Acyl-CoA Dehydrogenases (ACADs) and Enoyl-CoA Isomerases.* The phylogenetic tree illustrates the evolutionary relationships among selected ACADs and Enoyl-CoA Isomerases, constructed using Clustal Omega multiple sequence alignment[12]. The evolutionary separation between ACADs and Enoyl-CoA Isomerases supports their non-overlapping catalytic roles. In particular ACAD9 (Uniprot ID Q9H845) and VLCAD (or ACADV, P49748) are closely related, reflecting their shared role in long-chain fatty acid β-oxidation. MCAD (or ACADM, P11310) and SCAD (or ACADS, P16219) form a separate clade, corresponding to their function in medium- and short-chain fatty acid oxidation, respectively. LCAD (or ACADL, P28330) and Isobutyryl-CoA Dehydrogenase (or ACAD8, Q9UKU7) also form a distinct subgroup, likely reflecting adaptations to substrate specificity. Enoyl-CoA Isomerases (ECI1, ECI2/ECH1, and ECHM) form a distinct evolutionary branch, separate from the ACAD family. ECI1 (P42126) and ECH1 (Q13011) are closely related, supporting their functional role in enoyl-CoA double-bond migration during β-oxidation. ECHM (ECHS1, P30084) clusters with enoyl-CoA isomerases but remains functionally distinct as a hydratase. The scale bar (top) represents evolutionary distance, with values indicating substitutions per site.

Molecular ion<br>M = C16:0-CoA	m/z	Mass (Da)<br>by replacing Li/Na with H
$[M]^+$	1006.3306	1005.3226
$[M(Li)]^+$	1012.3741	1005.4341
$[M(Na)]^+$	1028.3301	1005.3403
$[M(Li)(Na)]^+$	1034.3615	1005.4397
$[M(Na)_2]^+$	1050.3324	1005.3608
$[M(Li)(Na)_2]^+$	1056.3176	1005.4140
$[M(Na)_3]^+$	1072.2981	1005.3447
$[M(Li)(Na)_3]^+$	1078.3453	1005.4599
$[M(Na)_4]^+$	1094.2346	1005.2994

Table S1. *m/z values of the C16:0-CoA mass spectrum* (**Figure S3 B**).

**Molecular ion**<br>**M = C16:0-CoA**<br>**P = (*2E*)-C16:1-CoA, (*3E*)-C16:1-CoA and (*3Z*)-C16:1-CoA**	**m/z**	**Mass (Da)**<br>**by replacing Li/Na with H**
[P(Na)]$^+$	1026.3020	1003.3122
[P(Li)(Na)]$^+$	1032.3215	1003.4095
[P(Na)$_2$]$^+$	1048.2876	1003.3356
[P(Li)(Na)$_2$]$^+$	1054.3048	1003.4012
[P(Na)$_3$]$^+$	1070.2655	1003.3121
[P(Li)(Na)$_3$]$^+$	1076.2917	1003.4063
[P(Na)$_4$]$^+$	1092.2551	1003.3199
[M(Na)]$^+$	1028.3197	1005.3299
[M(Li)(Na)]$^+$	1034.3319	1005.4101
[M(Na)$_2$]$^+$	1050.3003	1005.3287
[M(Li)(Na)$_2$]$^+$	1056.3099	1005.4063
[M(Na)$_3$]$^+$	1072.2858	1005.3324
[M(Li)(Na)$_3$]$^+$	1078.2908	1005.4054
[M(Na)$_4$]$^+$	1094.2690	1005.3338

Table S2. *m/z values of C16:0-CoA in presence of* ***ACAD9*** (**Figure S5**). The exacts mass for the C16:0-CoA is (1005.36±0.04)Da, the exact mass for the dehydrogenation products (2*E*)- C16:1-CoA, (3*E*)-C16:1-CoA and (3*Z*)-C16:1-CoA is aways (1003.36±0.05) Da.

**Molecular ion**<br>**M = C16:0-CoA**<br>**P = (2*E*)-C16:1-CoA, (3*E*)-C16:1-CoA and (3*Z*)-C16:1-CoA**	**m/z**	**Mass (Da)**<br>**by replacing Li/Na with H**
$[P(Na)]^{+}$	1026.3119	1003.3221
$[P(Li)(Na)]^{+}$	1032.3182	1003.3964
$[P(Na)_2]^{+}$	1048.2843	1003.3127
$[P(Li)(Na)_2]^{+}$	1054.3015	1003.3979
$[P(Na)_3]^{+}$	1070.2755	1003.3221
$[P(Li)(Na)_3]^{+}$	1076.2883	1003.4029
$[P(Na)_4]^{+}$	1092.2517	1003.3165
$[M(Na)]^{+}$	1028.3164	1005.3266
$[M(Li)(Na)]^{+}$	1034.3287	1005.4069
$[M(Na)_2]^{+}$	1050.2970	1005.3254
$[M(Li)(Na)_2]^{+}$	1056.3066	1005.4030
$[M(Na)_3]^{+}$	1072.2823	1005.3289
$[M(Li)(Na)_3]^{+}$	1078.2874	1005.4020
$[M(Na)_4]^{+}$	1094.2655	1005.3303

Table S3. *m/z values of C16:0-CoA in presence of* ***VLCAD*** (**Figure S6**). The exacts mass for the C16:0-CoA is (1005.36±0.04)Da, the exact mass for the dehydrogenation products (2*E*)-C16:1-CoA, (3*E*)-C16:1-CoA and (3*Z*)-C16:1-CoA is (1003.35±0.04)Da.

**Molecular ion M = (9Z)-C16:1-CoA**	**m/z**	**Mass (Da) by replacing Li/Na with H**
$[M]^-$	1002.316	1003.324
$[M(Li)]^-$	1008.326	1003.402
$[M(Na)]^-$	1024.302	1003.328
$[M(Li)(Na)]^-$	1030.312	1003.406
$[M(Na)_2]^-$	1046.287	1003.331

Table S4. *m/z values of the (9Z)-C16:1-CoA mass spectrum* (**Figure S9 B**).

**Molecular ion**<br>**M = (9*Z*)-C16:1-CoA**<br>**P = (2,9*Z*)-C16:2-CoA and (3,9*Z*)-C16:2-CoA**	**m/z**	**Mass (Da)**<br>**by replacing Li/Na with H**
$[P]^-$	1000.301	1001.309
$[P(Na)]^-$	1022.288	1001.314
$[P(Na)_2]^-$	1044.263	1001.307
$[M]^-$	1002.322	1003.330
$[M(Na)]^-$	1024.305	1003.331
$[M(Na)_2]^-$	1046.275	1003.319

Table S5. *m/z values of (9Z)-C16:1-CoA in presence of* ***ACAD9*** (**Figure S11**). The exacts mass for the (9*Z*)-C16:1-CoA is (1003.327±0.007)Da, the exact mass for the dehydrogenation products (2,9*Z*)-C16:2-CoA and (3,9*Z*)-C16:2-CoA is (1001.310±0.004)Da.

Palmitoyl Coenzyme A (C16:0-CoA)

Palmitic acid

^{1}H – (ppm)

16_(CH_3)	(CH_2)$_{11}$	4γ_(CH_2)	3β_(CH_2)	2α_(CH_2)
0.84 (t, J =7.23 Hz, 3H)	1.18-1.28 (m, 22H)	1.22 (m, 2H)	1.57 (m, 2H)	2.57 (t, J =7.72 Hz, 2H)

^{13}C – (ppm)

	15_(CH_2)	14_(CH_2)	(CH_2)$_9$			
15.50	24.09	33.22	28.89-31.36	31.92	27.28	45.5

Cysteamine

^{1}H – (ppm)

1_(CH_2)	2_(CH_2)
2.98 (t, J = 6.54Hz, 2H)	3.33 (t, J = 6.66Hz, 2H)

^{13}C – (ppm)

30.14	40.61

Pantothenic acid

^{1}H – (ppm)

1_(CH_2)	2_(CH_2)	3_(CH)	4_(CH_3)	5_(CH_3)	6_(CH)	7_(CH)
2.41 (t, J =7.08Hz, 2H)	3.44 (t, J = 7.02, 2H)	4.02 (s, 1H)	0.73 (s, 3H)	0.87 (s, 3H)	3.54 (m, 1H)	3.83 (m, 1H)

^{13}C – (ppm)

37.56	37.56	76.10	20.15	22.79	73.97	73.97

3'- phosphoadenosine - 5' - diphosphate

^{1}H – (ppm)

5_(CH_2)	4_(CH)	1_(CH)	6_(CH)	7_(CH)
4.22 (m, 2H)	4.56	6.15 (d, J = 6.46Hz, 2H)	8.55 (s, 1H)	8.24 (s, 1H)

^{13}C – (ppm)

67.64	85.97	88.46	141.96	155.02

Table S6 *^{1}H and ^{13}C NMR assignments for C16:0-CoA.*

Palmitoleoyl Coenzyme A ((9*Z*)-C16:1-CoA)

Palmitoleic acid							
^{1}H – (ppm)							
16_(CH$_3$)	(CH$_2$)$_3$	6/13_(CH$_2$), 4γ _(CH$_2$)	7/12_(CH$_2$)	8/11_(CH$_2$)	9/10_(CH)	3β _(CH$_2$)	2α_(CH$_2$)
0.83 (t, J =7.27 Hz, 3H)	1.21-1.24 (m)	1.22 (m)	1.27 (m)	1.98 (m, 4H)	5.41 (m)	1.57 (m, 2H)	2.57 (t, 7.39Hz)

Cysteamine	
^{1}H – (ppm)	
1_(CH$_2$)	2_(CH$_2$)
2.99 (t, J = 6.47Hz, 2H)	3.33 (t, J = 6.41Hz, 2H)

Pantothenic acid						
^{1}H – (ppm)						
1_(CH$_2$)	2_(CH$_2$)	3_(CH)	4_(CH$_3$)	5_(CH$_3$)	6_(CH)	7_(CH)
2.42 (t, J =6.91Hz, 2H)	3.44 (t, J = 6.97, 2H)	4.03 (s, 1H)	0.74 (s, 3H)	0.88 (s, 3H)	3.55 (m, 1H)	3.83 (m, 1H)

3'- phosphoadenosine - 5' - diphosphate				
^{1}H – (ppm)				
5_(CH$_2$)	4_(CH)	1_(CH)	6_(CH)	7_(CH)
4.23 (m, 1H)	4.57	6.16 (d, J = 6.74Hz, 1H)	8.55 (s, 1H)	8.25 (s, 1H)

Table S7 *^{1}H NMR assignments for (9Z)-C16:1-CoA.*

Palmitoyl Coenzyme A (C16:0-CoA) after incubation with VLCAD or ACAD9

Product 1: (2E)-Hexadecenoyl Coenzyme A ((2E)-C16:1-CoA)

(2E)-Hexadecenoic acid				
**^{1}H – (ppm)**				
2α_(CH)	3β_(CH)	4γ_(CH_2)	5_(CH_2)	6_(CH_2)
6.17 (d, J = 23 Hz)	6.94	2.18	1.40	1.21
**^{13}C – (ppm)**				
129.87	151.21	33.62	29.16	-

Product 2 and 3: (3E)-Hexadecenoyl Coenzyme A ((3*E*)-C16:1-CoA)and (3Z)-Hexadecenoyl Coenzyme A ((3Z)-C16:1-CoA)

(3E)-Hexadecenoic acid, (3Z)-Hexadecenoic acid					
**^{1}H – (ppm)**					
2α_(CH_2)	3β_(CH)	4γ_(CH)	5_(CH_2)	6_(CH_2)	7_(CH_2)
3.22	5.66	5.44	2.00	1.30	1.22
**^{13}C – (ppm)**					
48.73	139.58	122.50	33.63 (E), 28.62 (Z)	30.12	-

Table S8 *^{1}H and ^{13}C NMR assignments for C16:0-CoA after incubation with ACAD9 or VLCAD.*

Palmitoleoyl Coenzyme A ((9*Z*)-C16:1-CoA) after incubation with ACAD9

Product 1: (2,9Z)-Hexadecadienoyl Coenzyme A ((2,9Z)-C16:2-CoA)

(2,9Z)-Hexadecadienoic acid				
**^1H – (ppm)**				
2α_(CH)	3β_(CH)	4γ_(CH_2)	5_(CH_2)	6_(CH_2)
6.16	6.93	2.17	1.41	1.30

Product 2: (3,9Z)-Hexadecadienoyl Coenzyme A ((3,9Z)-C16:2-CoA)

(3,9Z)-Hexadecadienoic acid				
**^1H – (ppm)**				
2α_(CH_2)	3β_(CH)	4γ_(CH)	5_(CH_2)	6_(CH_2)
3.22	5.65	5.45	2.00	1.33

Tab S9 *^1H NMR assignments for (9Z)-C16:1-CoA after incubation with ACAD9.*

REFERENCES

1. Meng, E. C.; Goddard, T. D.; Pettersen, E. F.; Couch, G. S.; Pearson, Z. J.; Morris, J. H.; Ferrin, T. E., UCSF ChimeraX: Tools for structure building and analysis. *Protein Science* **2023,** *32* (11), e4792.
2. Jumper, J.; Evans, R.; Pritzel, A.; Green, T.; Figurnov, M.; Ronneberger, O.; Tunyasuvunakool, K.; Bates, R.; Žídek, A.; Potapenko, A.; Bridgland, A.; Meyer, C.; Kohl, S. A. A.; Ballard, A. J.; Cowie, A.; Romera-Paredes, B.; Nikolov, S.; Jain, R.; Adler, J.; Back, T.; Petersen, S.; Reiman, D.; Clancy, E.; Zielinski, M.; Steinegger, M.; Pacholska, M.; Berghammer, T.; Bodenstein, S.; Silver, D.; Vinyals, O.; Senior, A. W.; Kavukcuoglu, K.; Kohli, P.; Hassabis, D., Highly accurate protein structure prediction with AlphaFold. *Nature* **2021,** *596* (7873), 583-589.
3. McGregor, L.; Acajjaoui, S.; Desfosses, A.; Saïdi, M.; Bacia-Verloop, M.; Schwarz, J. J.; Juyoux, P.; von Velsen, J.; Bowler, M. W.; McCarthy, A. A.; Kandiah, E.; Gutsche, I.; Soler-Lopez, M., The assembly of the Mitochondrial Complex I Assembly complex uncovers a redox pathway coordination. *Nat Commun* **2023,** *14* (1), 8248.
4. Giachin, G.; Jessop, M.; Bouverot, R.; Acajjaoui, S.; Saidi, M.; Chretien, A.; Bacia-Verloop, M.; Signor, L.; Mas, P. J.; Favier, A.; Borel Meneroud, E.; Hons, M.; Hart, D. J.; Kandiah, E.; Boeri Erba, E.; Buisson, A.; Leonard, G.; Gutsche, I.; Soler-Lopez, M., Assembly of The Mitochondrial Complex I Assembly Complex Suggests a Regulatory Role for Deflavination. *Angew Chem Int Ed Engl* **2021,** *60* (9), 4689-4697.
5. Xia, C.; Lou, B.; Fu, Z.; Mohsen, A. W.; Shen, A. L.; Vockley, J.; Kim, J. P., Molecular mechanism of interactions between ACAD9 and binding partners in mitochondrial respiratory complex I assembly. *iScience* **2021,** *24* (10), 103153.
6. Zhang, Y.; Mohsen, A.-W.; Kochersperger, C.; Solo, K.; Schmidt, A. V.; Vockley, J.; Goetzman, E. S., An acyl-CoA dehydrogenase microplate activity assay using recombinant porcine electron transfer flavoprotein. *Analytical Biochemistry* **2019,** *581*, 113332.
7. Frerman, F. E.; Goodman, S. I., Fluorometric assay of acyl-CoA dehydrogenases in normal and mutant human fibroblasts. *Biochem Med* **1985,** *33* (1), 38-44.
8. Małecki, J.; Ho, A. Y.; Moen, A.; Dahl, H. A.; Falnes, P., Human METTL20 is a mitochondrial lysine methyltransferase that targets the β subunit of electron transfer flavoprotein (ETFβ) and modulates its activity. *J Biol Chem* **2015,** *290* (1), 423-34.
9. Kragelund, B. B.; Andersen, K. V.; Madsen, J. C.; Knudsen, J.; Poulsen, F. M., Three-dimensional Structure of the Complex between Acyl-Coenzyme A Binding Protein and Palmitoyl-Coenzyme A. *Journal of Molecular Biology* **1993,** *230* (4), 1260-1277.
10. Lerche, M. H.; Kragelund, B. B.; Bech, L. M.; Poulsen, F. M., Barley lipid-transfer protein complexed with palmitoyl CoA: the structure reveals a hydrophobic binding site that can expand to fit both large and small lipid-like ligands. *Structure* **1997,** *5* (2), 291-306.
11. Wishart, D. S.; Guo, A.; Oler, E.; Wang, F.; Anjum, A.; Peters, H.; Dizon, R.; Sayeeda, Z.; Tian, S.; Lee, Brian L.; Berjanskii, M.; Mah, R.; Yamamoto, M.; Jovel, J.; Torres-Calzada, C.; Hiebert-Giesbrecht, M.; Lui, Vicki W.; Varshavi, D.; Varshavi, D.; Allen, D.; Arndt, D.; Khetarpal, N.; Sivakumaran, A.; Harford, K.; Sanford, S.; Yee, K.; Cao, X.; Budinski, Z.; Liigand, J.; Zhang, L.; Zheng, J.; Mandal, R.; Karu, N.; Dambrova, M.; Schiöth, Helgi B.; Greiner, R.; Gautam, V., HMDB 5.0: the Human Metabolome Database for 2022. *Nucleic Acids Research* **2021,** *50* (D1), D622-D631.
12. Madeira, F.; Madhusoodanan, N.; Lee, J.; Eusebi, A.; Niewielska, A.; Tivey, A. R. N.; Lopez, R.; Butcher, S., The EMBL-EBI Job Dispatcher sequence analysis tools framework in 2024. *Nucleic Acids Research* **2024,** *52* (W1), W521-W525.
